# Room temperature synthesis of biodiesel using sulfonated graphitic carbon nitride

**DOI:** 10.1038/srep39387

**Published:** 2016-12-19

**Authors:** R. B. Nasir Baig, Sanny Verma, Mallikarjuna N. Nadagouda, Rajender S. Varma

**Affiliations:** 1 Sustainable Technology Division, National Risk Management Research Laboratory, U. S. Environmental Protection Agency, MS 443, Cincinnati, Ohio 45268, USA; 2 WQMB, WSWRD, National Risk Management Research Laboratory, U. S. Environmental Protection Agency, Cincinnati, Ohio 45268, USA

## Abstract

Sulfonation of graphitic carbon nitride (g-CN) affords a polar and strongly acidic catalyst, Sg-CN, which displays unprecedented reactivity and selectivity in biodiesel synthesis and esterification reactions at room temperature.

The Energy is the key driving force for the habitants on the planet and is essential for sustaining transportation, industrialization, technological advancement and daily needs. The most common source to meet the energy demand is fossil-based petroleum^1^,^2^, although technological advances have reduced the immediate threat which might have occurred due to its scarcity. Adequate supplies of petroleum are available which is reflected in the steady fall in the price of petroleum products. The extensive use of petroleum in the transportation and industrial sector^3^,^4^ coupled with burning of huge amount of fossil fuels culminate in the release of copious amounts of toxic gases namely oxides of sulfur, carbon and nitrogen^5^,^6^. Environmental and air pollution is an immediate threat to human health emanating from the consumption of fossil fuels^7^,^8^. Air contamination by sulfur and nitrogenous gases mainly originate from impurities present in petroleum products, thereby contributing to global warming. Continued explorations to identify safer alternatives to substitute for the petroleum products is driven largely due to growing concern over the change in an ecological system^9^. Benign options are sought that are devoid of heteroatom impurities^10^.

Biodiesel has been recognized as a greener alternative to petroleum-based products^11^,^12^,^13^ and can be synthesized from the fat (a waste product in the meat processing industry) and a variety of vegetable oils. The fatty acids in fat and vegetable oils can be converted into biodiesel *via* esterification. Several pathways have been identified to convert fatty acids to biodiesel using acid catalysts^14^,^15^. The homogeneous acid catalysts applied in the synthesis of biodiesel carry the burden of tedious purification processes which directly impacts production costs^16^. Most acidic heterogeneous catalysts entail the use of toxic transition metals^17^, metal oxides^18^,^19^,^20^, metal organic frame work^21^ and metal nano-particles^22^. Porous carbon, polar silica and petroleum polymers have also been utilized in the esterification of fatty acids^23^,^24^,^25^,^26^,^27^,^28^,^29^. The main drawback of these catalysts has been high reaction temperatures, use of a hazardous support and the cost of the product. Often the catalyst is costlier then the product itself.

Engaged in the development of sustainable methods and converting waste products into valuable chemicals^30^,^31^,^32^,^33^,^34^, herein, we report the use of sulfonated graphitic carbon nitride (Sg-CN) as a benign and cost effective organo-sulfonated heterogeneous acid catalyst for the synthesis of biodiesel.

## Synthesis and characterization of catalyst

**Figure 1:**
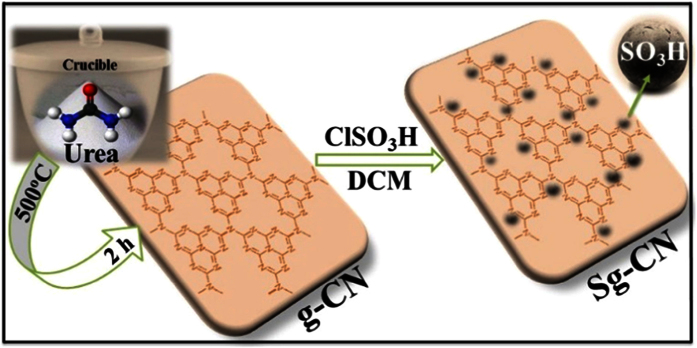
Synthesis of sulfonated graphitic carbon nitride (Sg-CN).

The Sulfonated graphitic carbon nitride (Sg-CN) has been designed and synthesized *via* sequential calcination^33^,^34^,^35^ and sulfonation of urea (Fig. 1).

**Figure 2:**
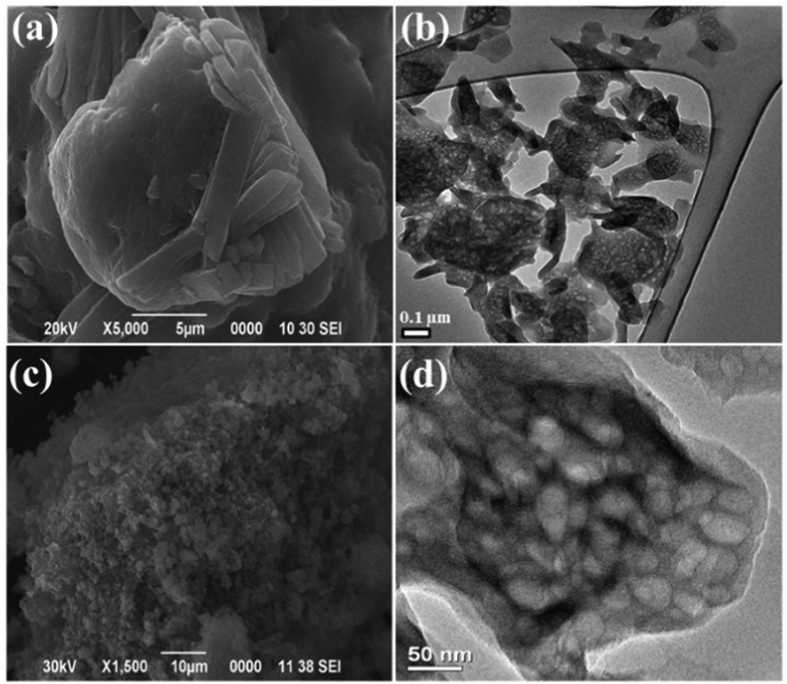
(**a**) SEM image of Sg-CN; (**b**) TEM image of Sg-CN. (**c**) SEM image of g-CN; (**d**) TEM image of g-CN.

The Sg-CN catalyst was characterized using scanning electron microscope (SEM), transmission electron microscope (TEM), X-ray diffraction (XRD), Fourier transform infrared spectroscopy (FT-IR), and solid-state nuclear magnetic resonance (^13^C-NMR). The SEM analysis clearly indicates the incorporation of sulfonic group in the graphitic carbon nitride framework; change in morphology of g-CN after sulfonation was also discerned (Fig. 2).

**Figure 3:**
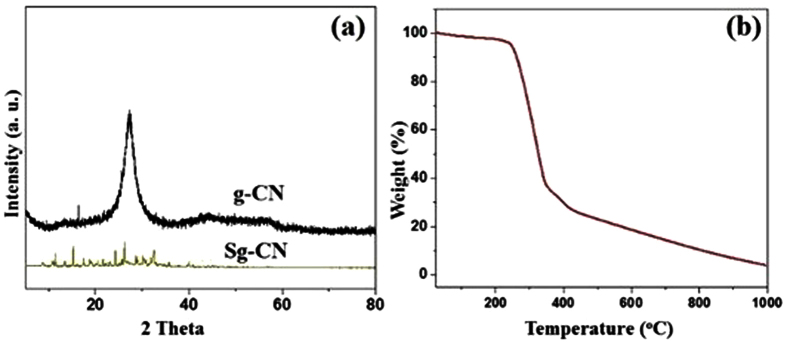
(**a**) XRD spectra of g-CN and Sg-CN; (**b**) TGA analysis of Sg-CN.

The examination of Sg-CN and starting material graphitic carbon nitride (g-CN) reveals the crystalline nature of Sg-CN which is reaffirmed by comparing the XRD pattern of Sg-CN and g-CN (Fig. 3a). The stability of the catalyst and temperature tolerance have been studied using thermogravimetric analysis (TGA), which confirms that the synthesized sulfonated graphitic carbon nitride is stable up 250 °C (Fig. 3b). The change in functional group and electronic behavior has been studied using FTIR and solid state ^13^C-NMR. Juxtapose of FT-IR and ^13^C-NMR spectra of g-CN and Sg-CN corroborate the presence of essential functionality differences and electronic nature (See Supplementary) as evidenced by the peak at 1200 cm^−1^ a characteristic signal for sulfonated graphitic carbon nitride (see Supplementary, Fig. S4) which is confirmed by comparative analysis of ^13^C-NMR of g-CN and Sg-CN (see Supplementary, Fig. S5) The BET surface analysis of g-CN (35.42 m^2^/g, see Supplementary, Fig. S5 and Fig. S6) and Sg-CN (10.04 m^2^/g, see Supplementary, Fig. S7 and Fig. S8) also confirmed the immobilization of sulfonic group. There is sharp decline in the surface area after the immobilization which is may be due to the creation of ionic character on the nitrogenous framework of g-CN culminating in the better interlayer attraction in graphitic carbon nitride.

The concentration of sulfur was determined using elemental analysis which corresponds to the 5.47 mmol/g of the catalyst, which is equivalent to the acid concentration in Sg-CN. The acid strength is expected to be higher due anticipated positive charge developed on the nitrogenous framework after the attachment of -SO_3_H.

## Results and Discussion

**Figure 4:**
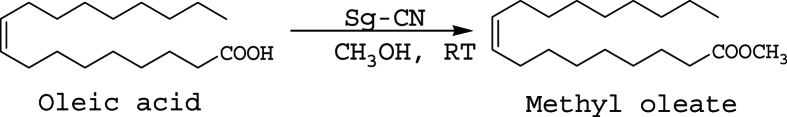
Esterification of oleic acid.

The application of Sg-CN was explored in the synthesis of biodiesel *via* the esterification of fatty acids. Oleic acid was used as a model substrate to optimize of reaction conditions (Fig. 4). Initially, one gram of oleic acid was treated with 100 mg of Sg-CN in methanol at room temperature (Table 1, entry 1) and the reaction was monitored using GC-MS at regular time intervals of time. Complete conversion of oleic acid to the corresponding methyl ester occurred within 4 hours. The most enthusing observation was the product purity attained after a simple decantation and distillation of reaction mixture. Experiments were conducted then to determine the optimum catalyst charge required for this efficient fatty acid esterification. Accordingly, oleic acid was treated with 75 mg, 50 mg, 25 mg, 10 mg and 5 mg of Sg-CN (Table 1, entries 2–5). Complete conversion of oleic acid to methyl oleate was discerned again (Table 1, entries 2–4) whereas the reaction with 10 mg of Sg-CN required overnight stirring for the completion of esterification at room temperature. However, further reduction in Sg-CN quantity does not allow the reaction to be completed even after 24 hours of stirring (Table 1, entry 6). A control experiment with pure support g-CN has been performed under similar conditions; even a trace of the product formation after 24 hours was not discernible. After finding optimum catalyst loading of Sg-CN, it was imperative to compare our results with the reported acid catalyst. We were pleasantly surprised to see that Sg-CN completed the reaction within 4 h at room temperature which was not precedence in earlier reports (Table 2)^13^,^36^,^37^,^38^,^39^

After establishing the optimum catalyst charge required for efficient esterification, a broader scope of the reaction was explored deploying a wide range of fatty acids and their analogues for the esterification and biodiesel production (Table 3). Most of the long chain fatty acids were efficiently converted into corresponding esters. The presence of unsaturation in the backbone does not affect the reaction outcome as all the acids were converted into corresponding esters almost in quantitative yield. Treatment of a bi-functional dicarboxylic acid with Sg-CN under similar conditions afforded the corresponding diester (Table 3, entry 5) although a relatively longer reaction time was required.

The transesterification reactions were also performed using ethyl benzoate and ethyl cinnamate using methanol as a solvent and Sg-CN as a catalyst (see Supplementary, Table S1). GCMS (see Supplementary) confirmed that the equilibrium shift completed towards the corresponding methyl esters. The solidification of the reaction towards the right may be due to higher concentration of methanol which is used as a reaction media in transesterification.

### Recycling and reusability of the Sg-CN

The stabilty and recyclability aspects of the catalyst were studied thereafter using oleic acid and Sg-CN. Upon reaction completion, the catalyst was seperated, washed with methanol, dried under vacuum and reused for the next set of reactants. The outcome of the recycling experiments authenticate that the catalyst can be reused up to 5 cycles without any loss in activity (see Supplementary, Fig. S2).

## Conclusion

A sulfonated graphitic carbon nitride (Sg-CN) has been synthesized *via* simple sulfonation and its application has been demonstrated in the efficient synthesis of biodiesel. The unique attribute of Sg-CN is its unprecedented reactivity which enables the esterification at room temperature, affording product that does not require any purification. The salient features of this catalyst include its relatively benign nature, easy accessibility, low cost and stability over several reaction cycles.

## Additional Information

**How to cite this article:** Baig, R. B. N. *et al*. Room temperature synthesis of biodiesel using sulfonated graphitic carbon nitride. *Sci. Rep.* **6**, 39387; doi: 10.1038/srep39387 (2016).

**Publisher's note:** Springer Nature remains neutral with regard to jurisdictional claims in published maps and institutional affiliations.

## Supplementary Material

Supplementary Information

## Figures and Tables

**Table 1:** Reaction optimization for the synthesis of biodiesel *via* esterification^a^.

Entry	Catalyst	Time	Conversion
1	100 mg	4 h	>99%
2	75 mg	4 h	>99%
3	50 mg	4 h	>99%
4	25 mg	4 h	>99%
5	10 mg	12 h	>99%
6	5 mg	24 h	<52%
7	25 mg	24	No reaction

Reaction Condition: Oleic acid (1.0 g), methanol (5.0 ml).

**Table 2:** Performances of various catalysts for the synthesis of biodiesel *via* esterification using oleic acid as the feedstock.

Entry	Catalyst	Performance	Reaction condition	Reference
1	Sulfonated porous organic co-polymer PDVTA-SO_3_H	92% fatty ester	10 h, 25 °C, 1:10; fatty acid : methanol, PDVTA-SO_3_H (10 mg)	13
2	Zr@UiO^−66^-NH_2_	88% fatty ester	20 h, 110 °C, over UiO-66-NH_2_(10 mol% Zr)	36
3	Lewis acidic ionic liquid catalyst (choline chloride·2ZnCl_2_)	98% fatty ester	12 h, 110 °C	37
4	Ammonium ceric sulfate	86.3% fatty ester	4 h, 75 °C	38
5	Phosphotungstic acid immobilized on the functionalized palygorskite	90% fatty ester	10 h, 100 °C	39
6	Sg-CN	>99%	4 h, room temperature	Present work

**Table 3:**
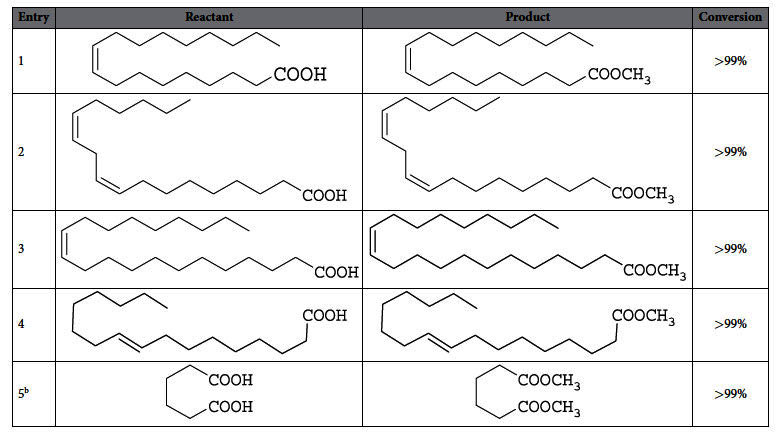
Sg-CN catalyzed esterification of fatty acids^a^.

^a^Reaction Condition: Fatty acid (1.0 g), methanol (5.0 ml), Sg-CN (25 mg), room temperature, 4 h;.

^b^Reaction was stirred for 8 h at room temperature.
