# Supplementary material for: Room temperature synthesis of biodiesel using sulfonated graphitic carbon nitride
Source: Sci Rep. 2016 Dec 19;6:39387. doi: 10.1038/srep39387 (PMC5172360; doi:10.1038/srep39387)
Supplement: Supplementary Information [file srep39387-s1.pdf]

Supporting Information

Room temperature synthesis of biodiesel using sulfonated graphitic carbon nitride

R. B. Nasir Baig[a]†, Sanny Verma[a]†, Mallikarjuna N. Nadagouda[b], Rajender S. Varma[a]*

[a]Sustainable Technology Division, National Risk Management Research Laboratory, U. S. Environmental Protection Agency, MS 443, Cincinnati, Ohio 45268, USA. Fax: 513- 569-7677; Tel: 513-487-2701. E-mail: varma.rajender@epa.gov

[b]WQMB, WSWRD, National Risk Management Research Laboratory, U. S. Environmental Protection Agency, MS 443, Cincinnati, Ohio 45268, USA

† Equal contribution

1. Synthesis of g-CN and Sg-CN

- **a) Synthesis of g-CN**
- **b) Synthesis of Sg-CN**

2. General procedure for the synthesis of biofuels

3. Sg-CN catalyzed transesterification reaction (Table S1)

4. Recycling of Sg-CN catalyst (S2)

5. FT-IR spectra of g-CN and Sg-CN (S3)

6. Solid state ^{13}CNMR of g-CN and Sg-CN (S4)

7. N_2 sorption isotherms of g-CN (S5)

8. Distribution of pore diameter of g-CN (S6)

9. N_2 sorption isotherms of Sg-CN (S7)

10. Distribution of pore diameter of Sg-CN (S8)

11. GC-MASS data of the product

12. ^{1}H and ^{13}C NMR of the product

1. Synthesis of g-CN and Sg-CN catalyst

a) Synthesis of g-CN

The pure urea obtained from Aldrich was calcined at 500 ^{o}C for 2 hours in a closed furnace. A pale yellow solid of pure graphitic carbon nitride (g-CN) was obtained and used without any further purification.

b) Synthesis of Sg-CN catalyst

Graphitic carbon nitride, g-CN (1.0 g) and dichloromethane (50 mL) were taken in a round bottom flask. Chlorosulfonic acid (0.5 mL) was added to the reaction mixture over the period of 10 min under continuous stirring which was continued for 3 hours. The resultant white solid was filtered off, washed with water, methanol and dried under vacuum at 50 °C. The Sg-CN catalyst was characterized by transmission electron microscopy (TEM), scanning electron microscopy (SEM), X-ray diffraction (XRD), Fourier transform infrared spectroscopy (FTIR), thermogravimetric analysis (TGA) and Brunauer, Emmett and Teller (BET) analysis.

2. General procedure for the synthesis of Biodiesel

A reaction tube equipped with a stir bar was charged with a fatty acid (1.0 g), catalyst (25 mg), and methanol (5 mL). The reaction mixture was stirred for 4 hours at room temperature. After completion of the reaction, the catalyst was recovered using a centrifuge and the product was isolated using solvent extraction.

3. Sg-CN catalyzed transesterification reaction

Table S1. Sg-CN catalyzed transestrification[a]

Entry	Reactant	Product	Conversion
1	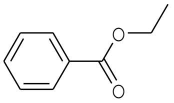	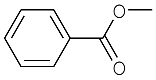	> 99%
2	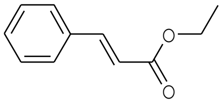	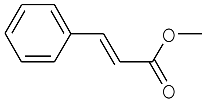	> 99%

a) Reaction condition: Ester (1.0 g), methanol (5.0 ml), Sg-CN (25 mg), room temperature, 4 h.

4. Recycling of the catalyst

After the completion of each reaction, the Sg-CN catalyst was recovered using a centrifuge, washed with water followed by methanol and dried under vacuum and used for a fresh set of reactants. It was observed that the catalyst remains active even after fifth cycle of the reaction.

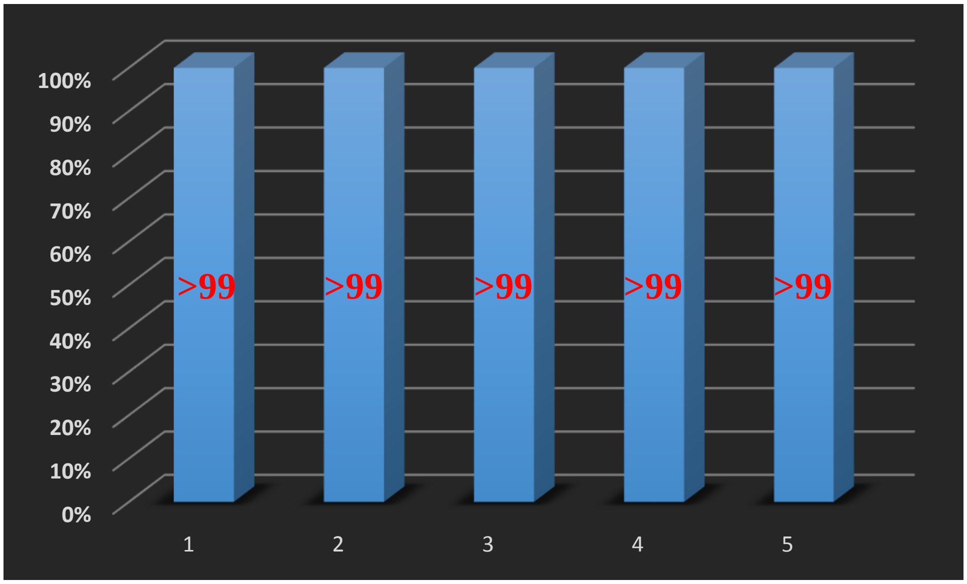

S2. Recycling of Sg-CN

5. **FTIR spectra of g-CN and Sg-CN**

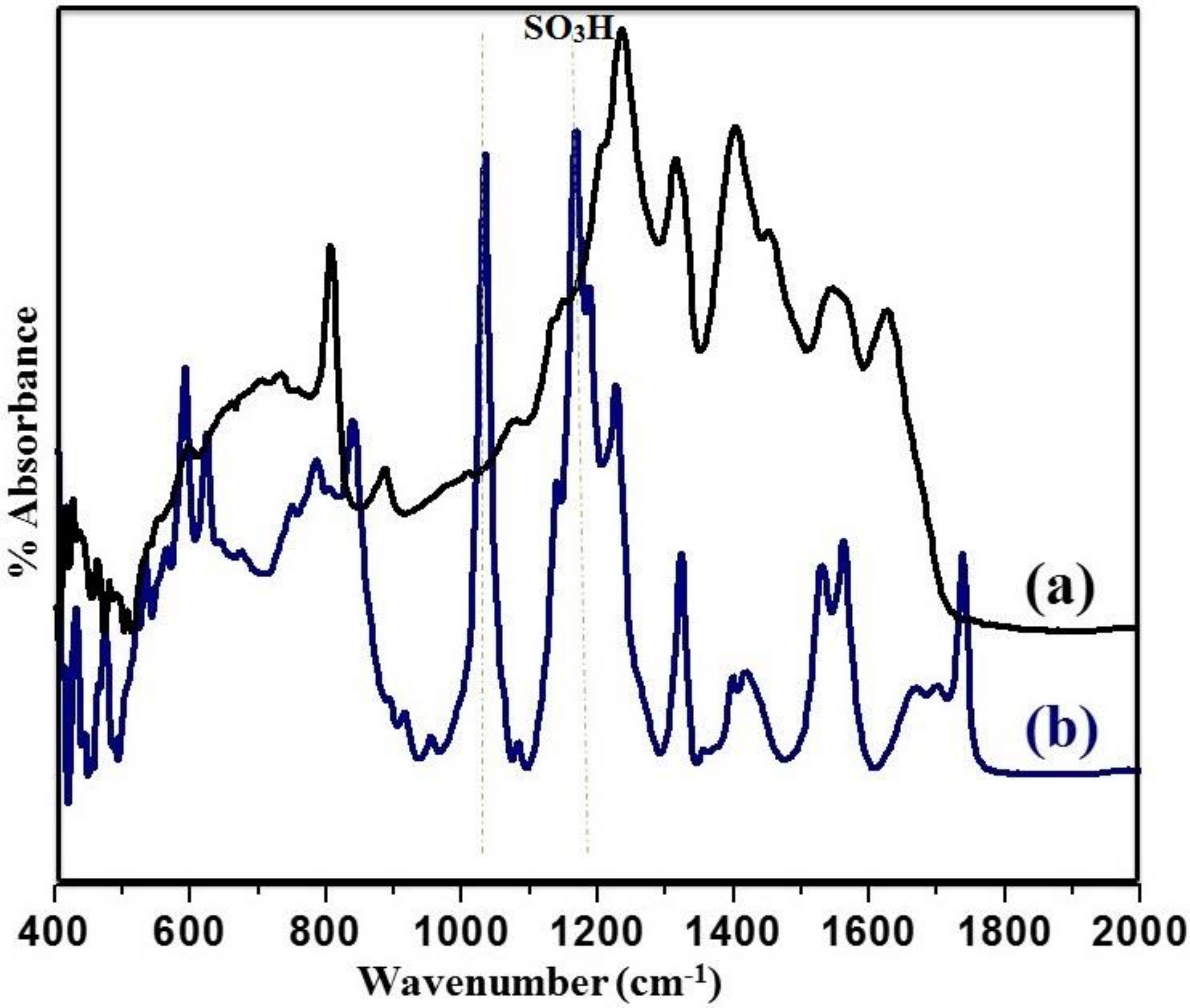

S3. FTIR spectra of a) g-CN and b) Sg-CN

6. Solid state ^{13}C NMR spectra of g-CN and Sg-CN

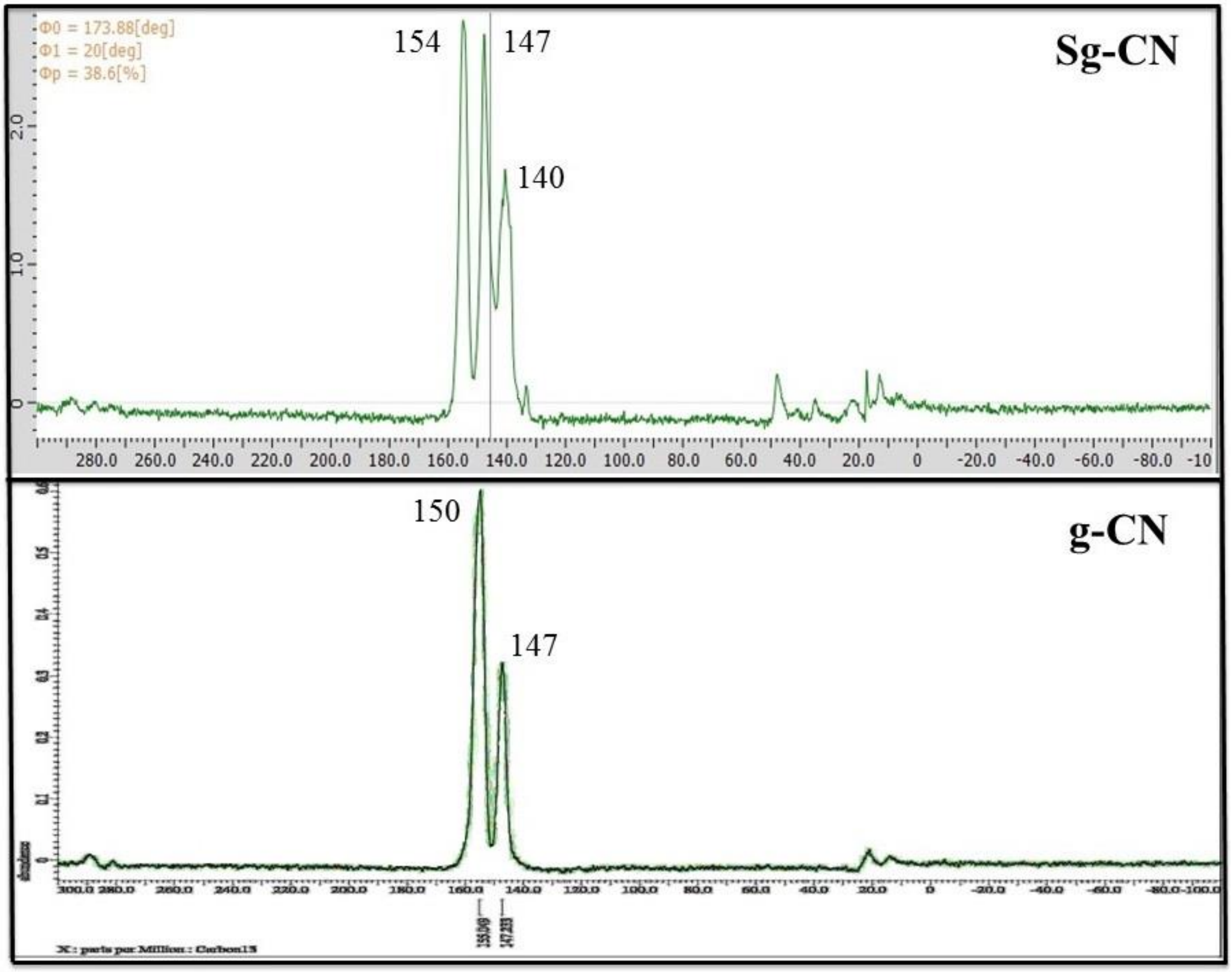

S4. Solid state ^{13}C NMR spectra of g-CN and Sg-CN

7. N_2 sorption isotherms of g-CN

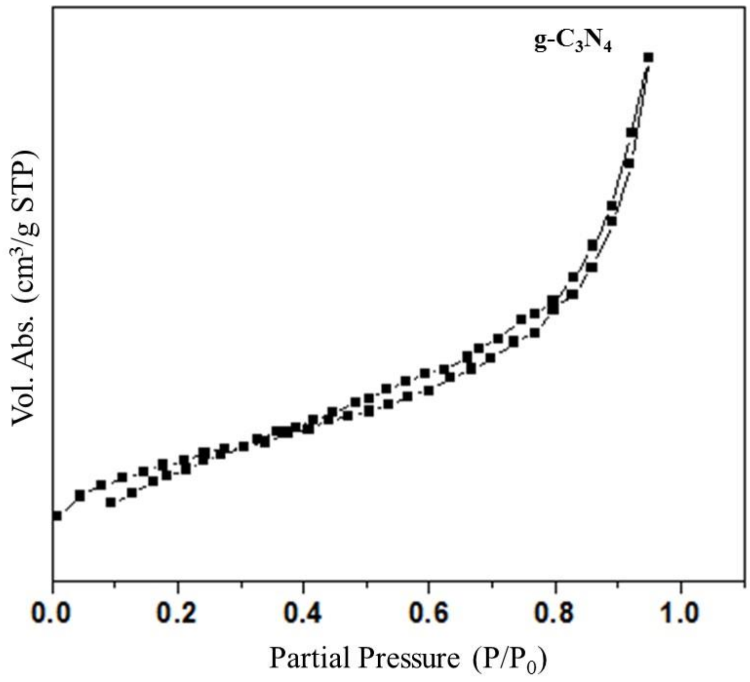

S5. N_2 sorption isotherms of g-CN

8. Distribution of pore diameter of g-CN

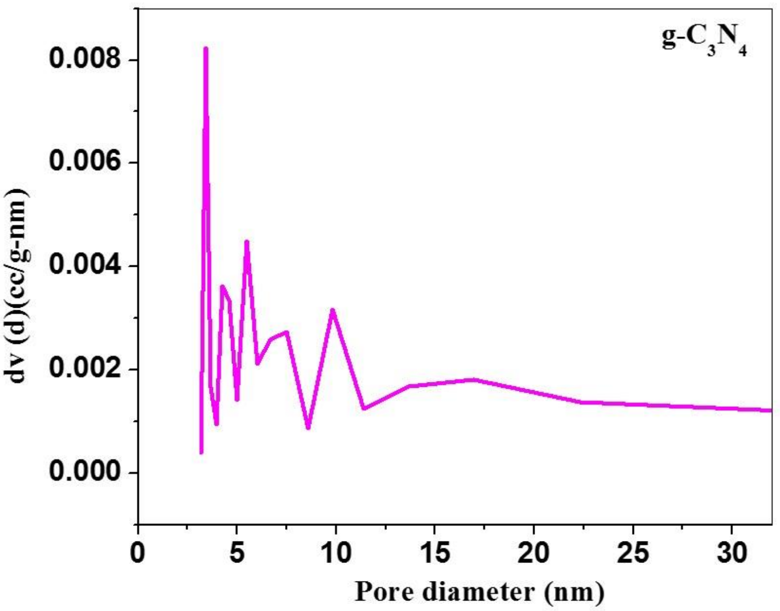

S6. Distribution of pore diameter of g-CN

9. N_2 sorption isotherms of Sg-CN

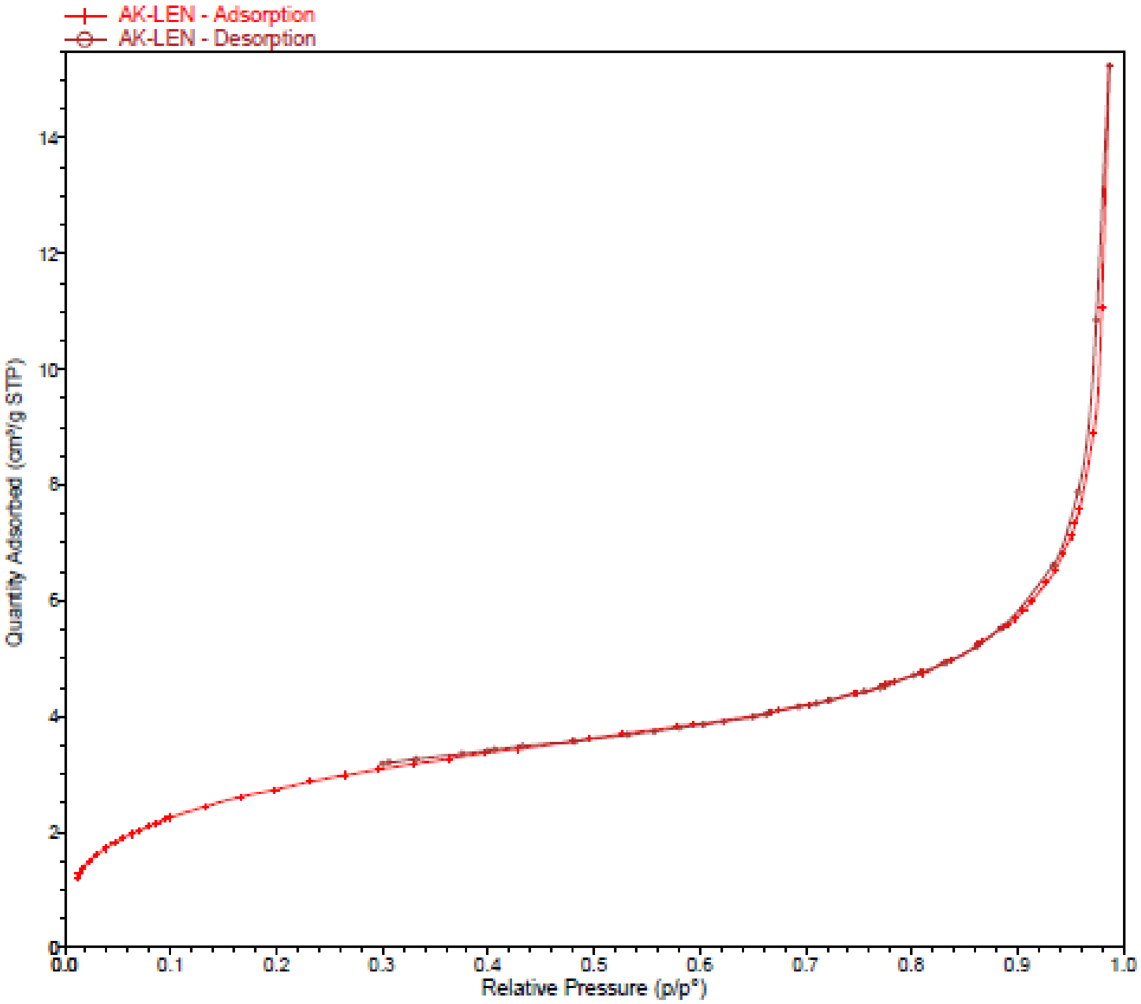

S7. N_2 sorption isotherms of Sg-CN

10. Distribution of pore diameter of Sg-CN

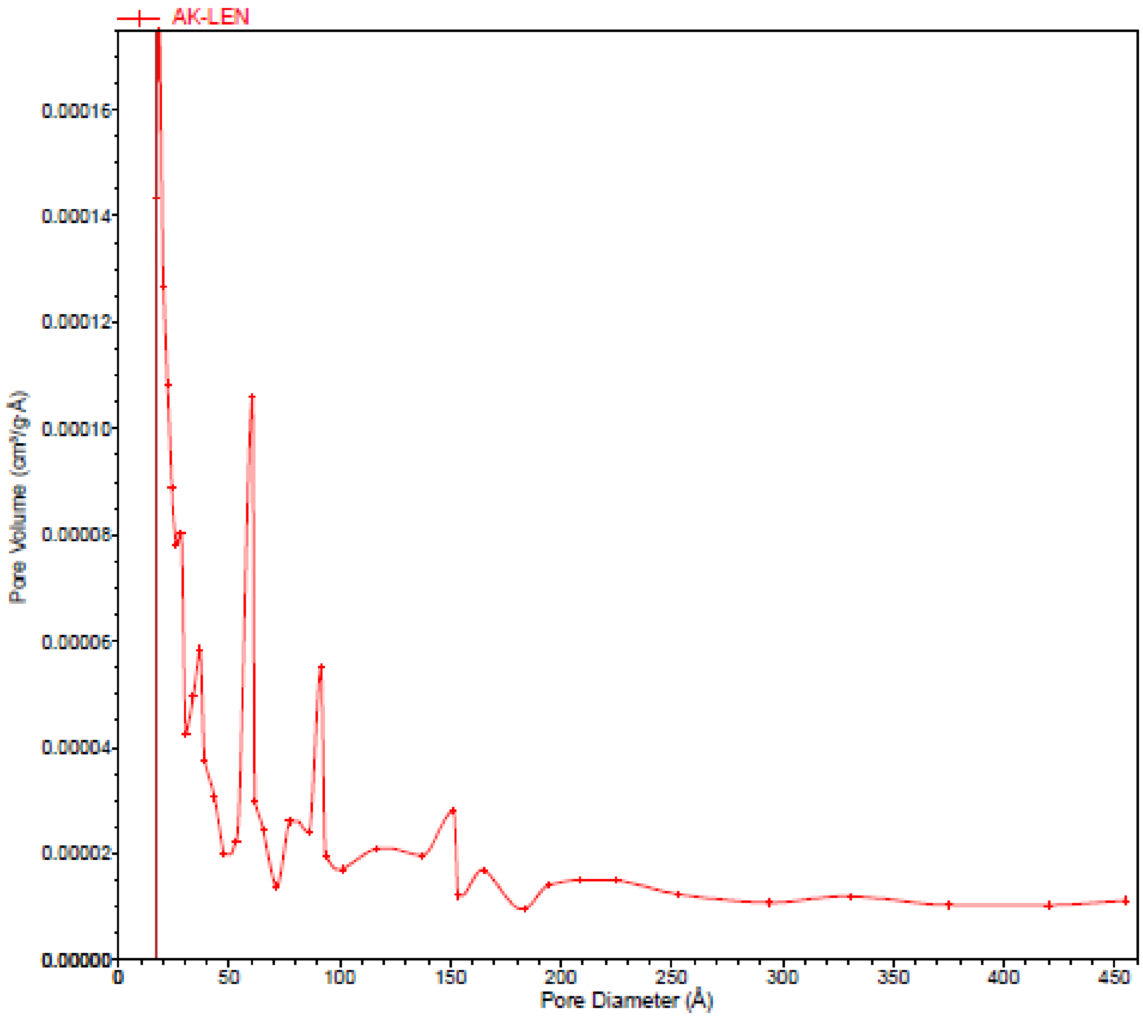

S8. Distribution of pore diameter of Sg-CN

11. GC-MASS data of the product

```
File        :C:\Sanny\Data\NS 149.D
Operator    : kt
Acquired    :  2 Dec 2015  10:01 am using AcqMethod SAMPLES- 20 MIN_20151015_A.M
Instrument :     Instrument #1
Sample Name: NS 149
Misc Info   :
Vial Number: 15
```

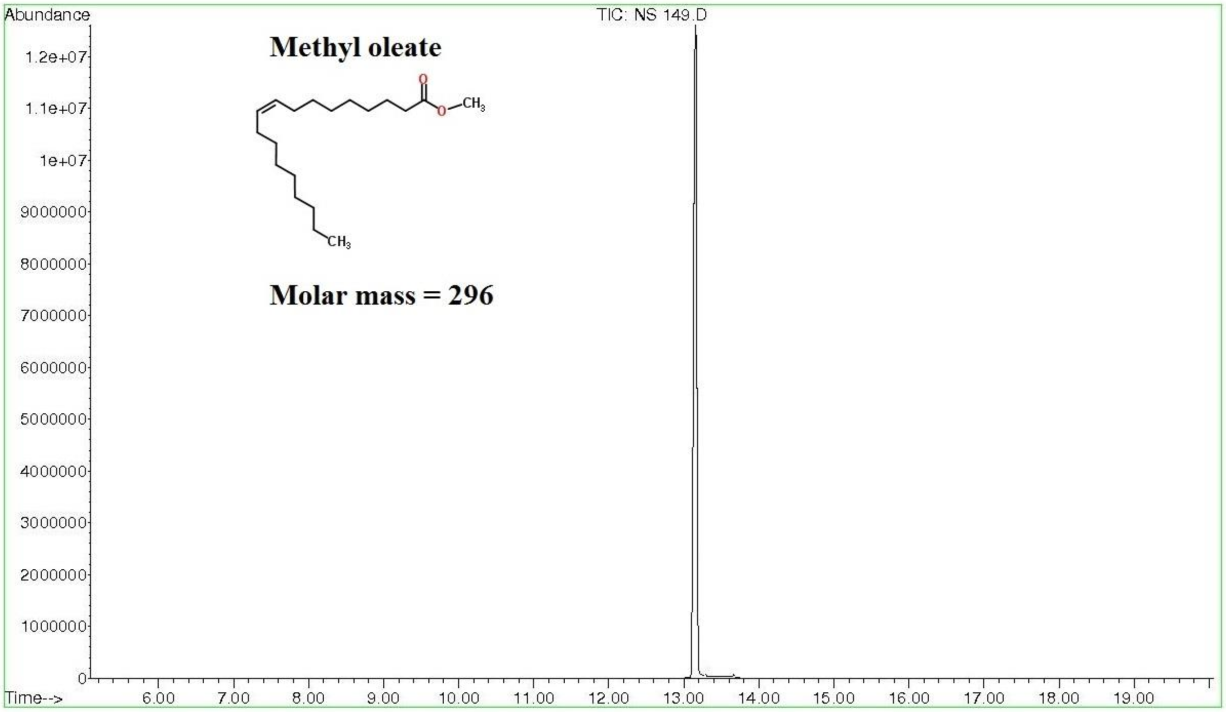

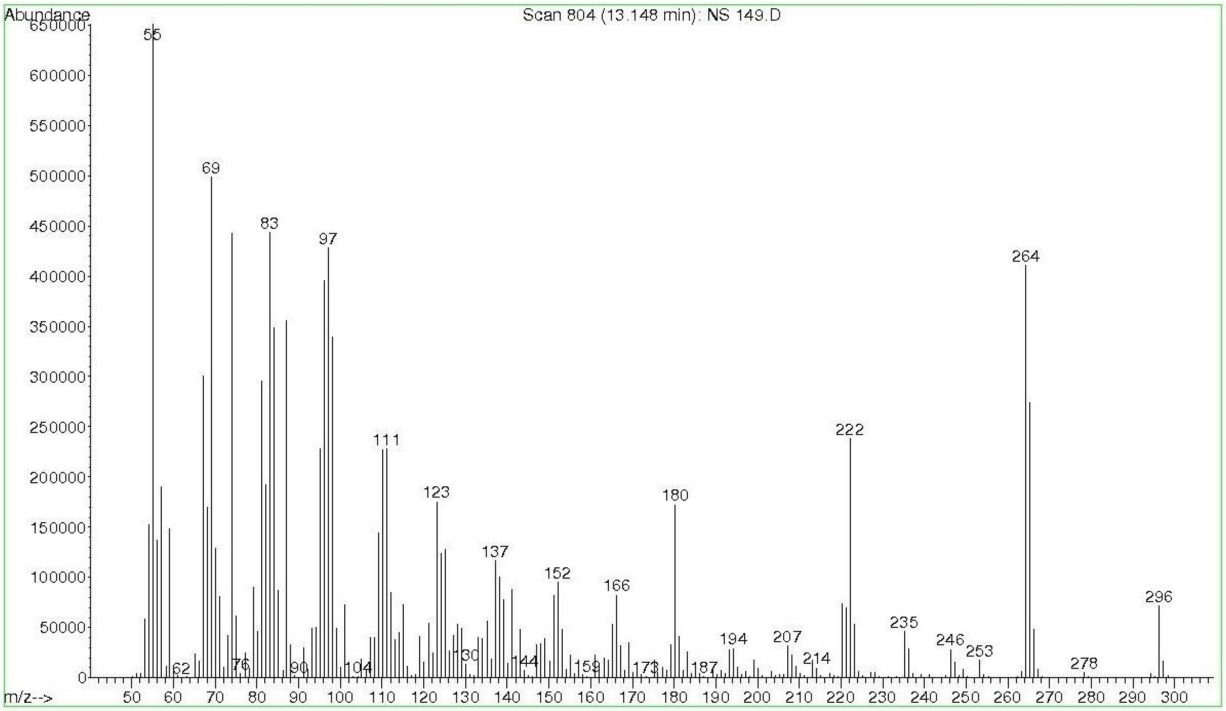

```
File        :C:\Sanny\Data\NS 148.D
Operator    : kt
Acquired    :  3 Dec 2015   1:43 pm using AcqMethod SAMPLES- 20 MIN_20151015_A.M
Instrument :     Instrument #1
Sample Name: NS 148
Misc Info   :
Vial Number: 13
```

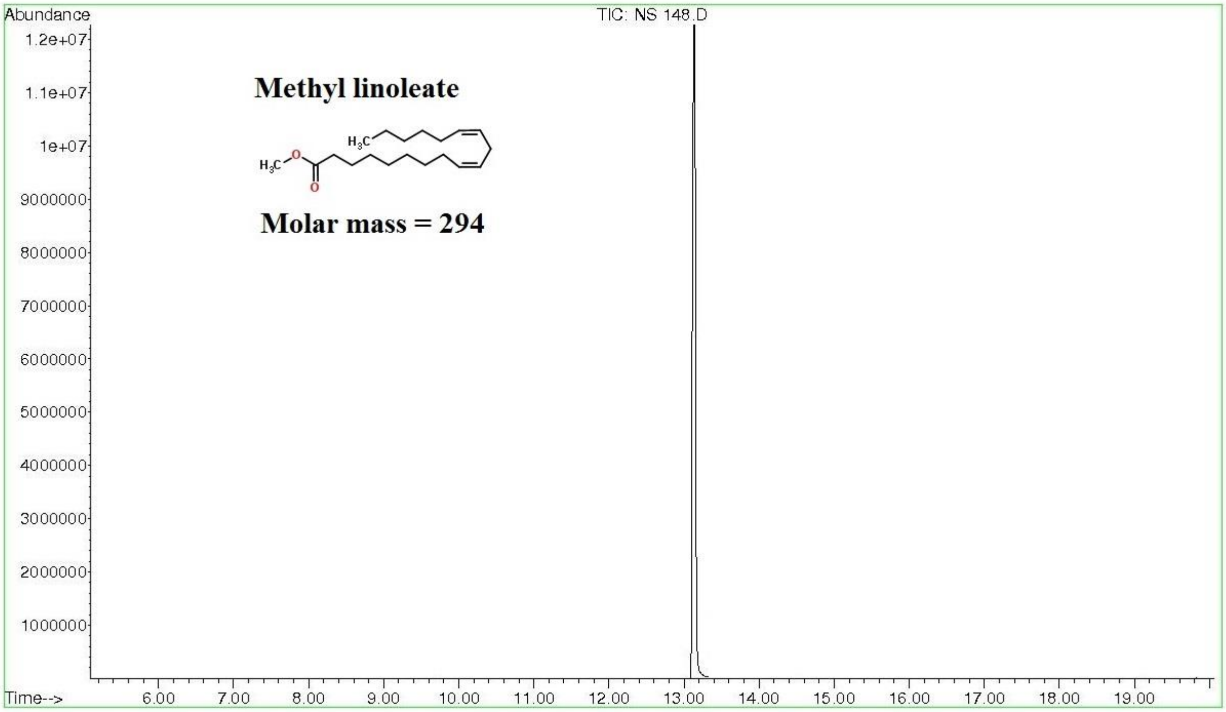

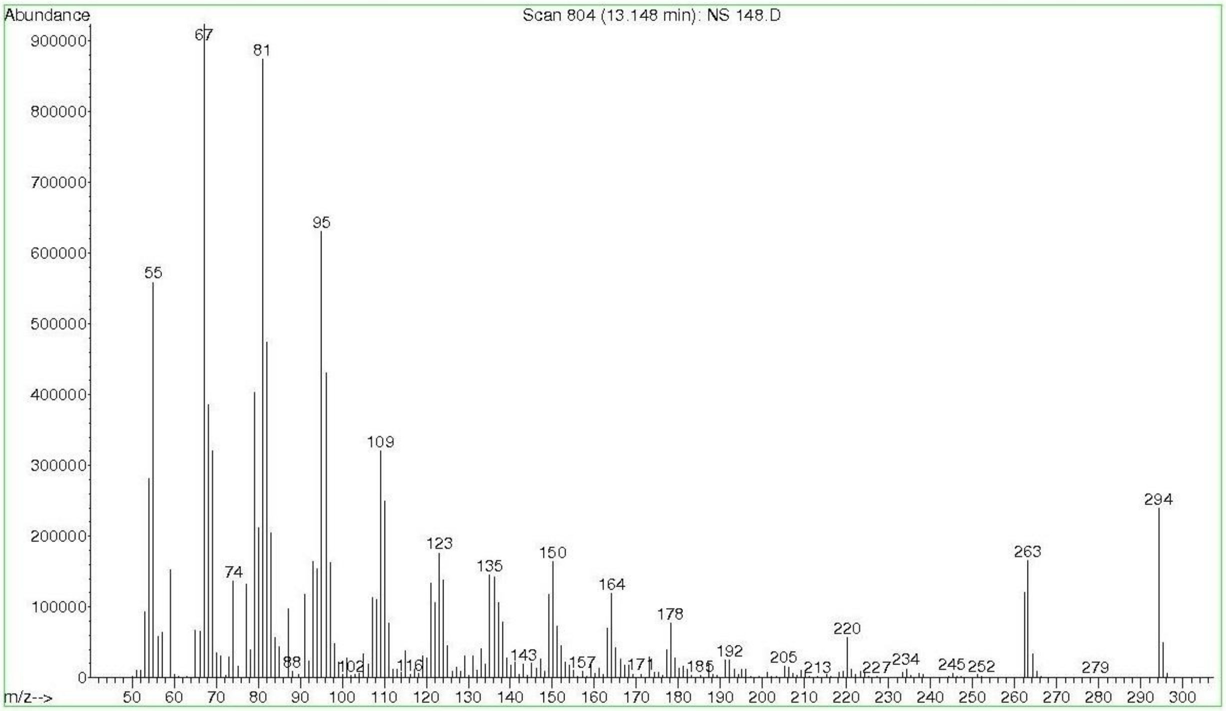

```
File        :C:\Sanny\Data\NS 147.D
Operator    : kt
Acquired    :  3 Dec 2015  12:34 pm using AcqMethod SAMPLES- 20 MIN_20151015_A.M
Instrument :     Instrument #1
Sample Name: NS 147
Misc Info  :
Vial Number: 12
```

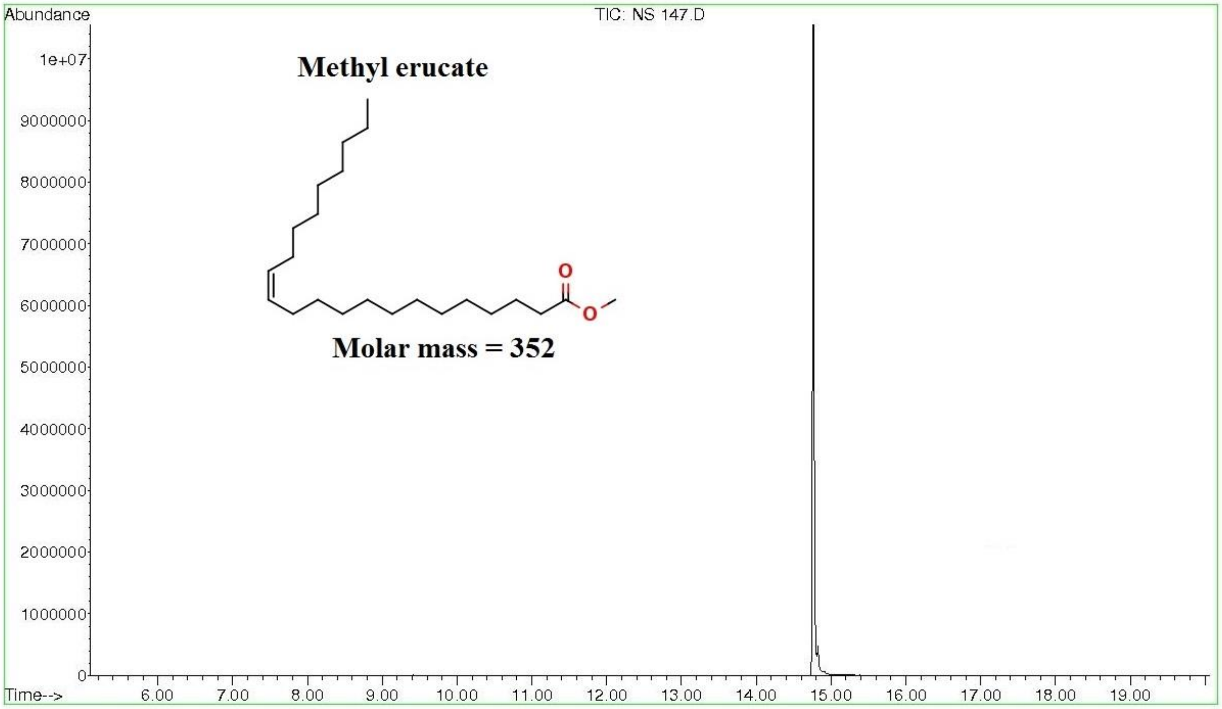

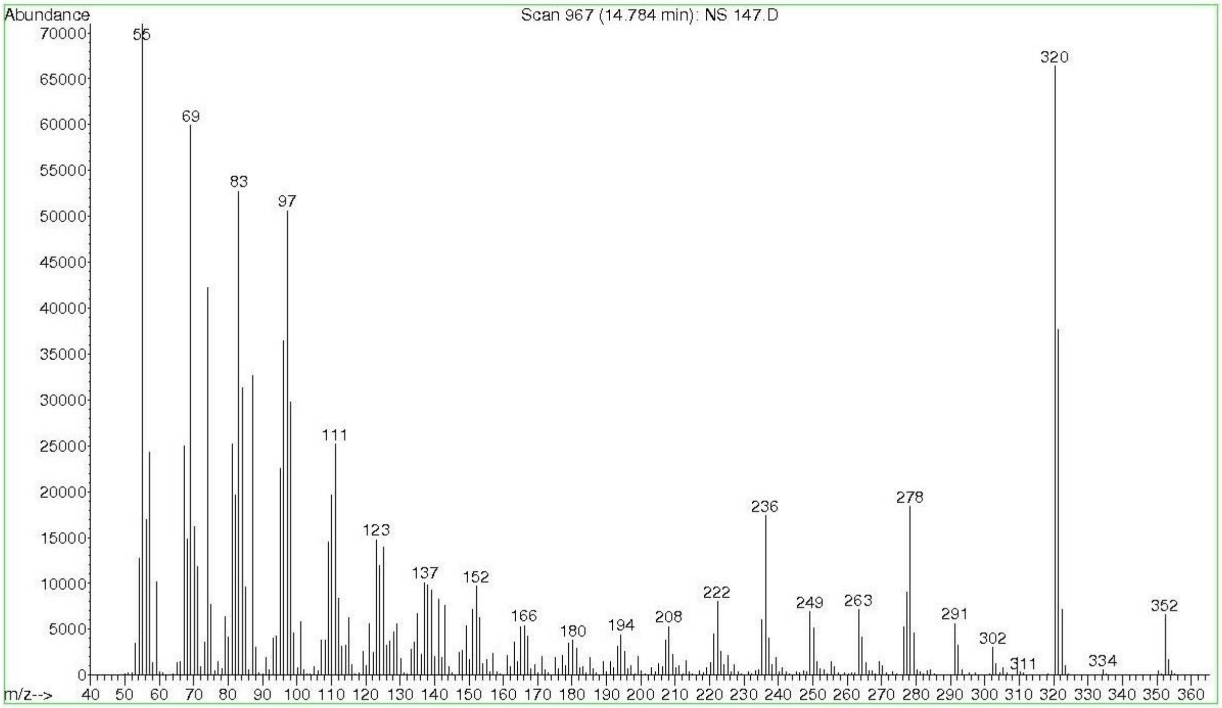

File   :C:\Sanny\Data\NS 146.D
Operator   : kt
Acquired   : 3 Dec 2015 12:00 pm using AcqMethod SAMPLES- 20 MIN_20151015_A.M
Instrument :   Instrument #1
Sample Name: NS 146
Misc Info :
Vial Number: 11

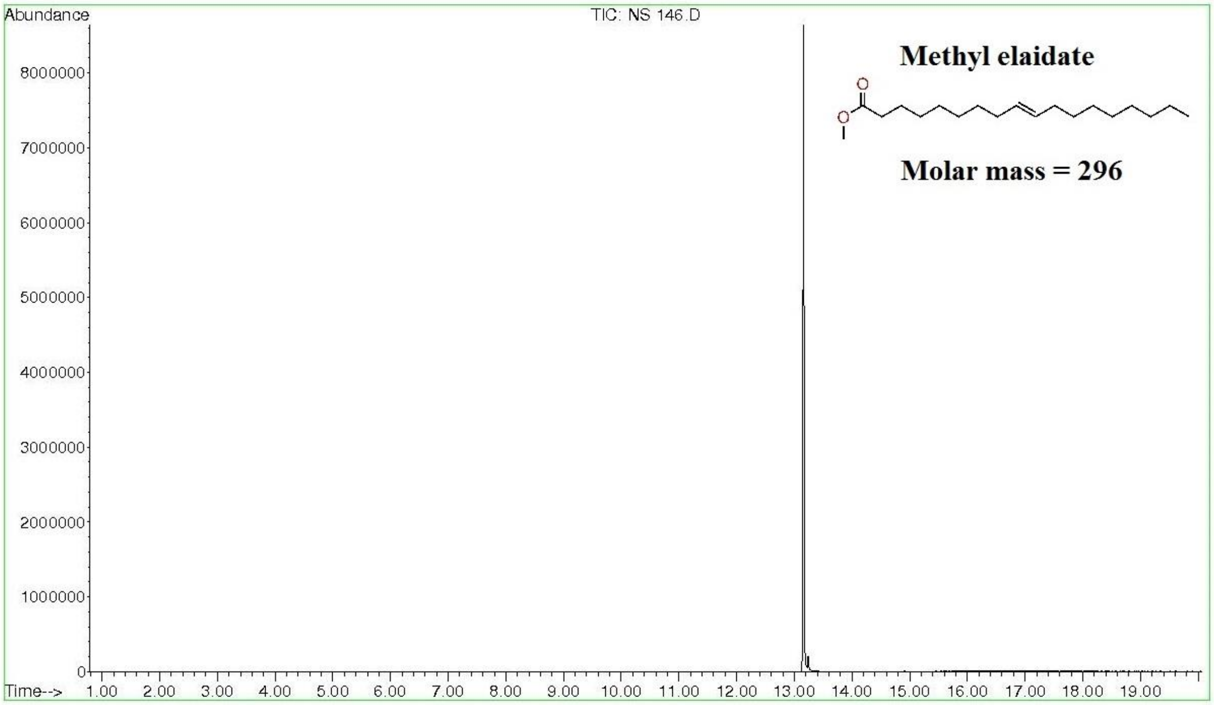

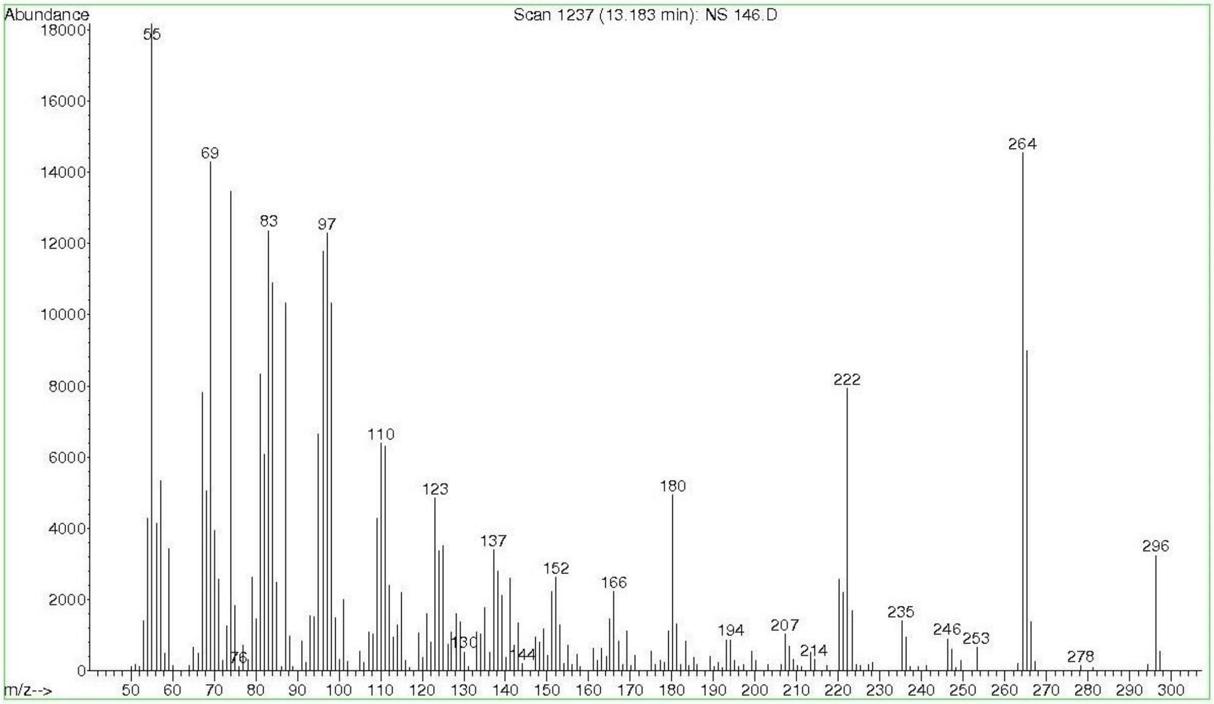

```
File        :C:\Sanny\Data\SS_56_Me.D
Operator    : kt
Acquired    : 30 Aug 2016  12:58 pm using AcqMethod SAMPLES- 20 MIN_20151015_A.M
Instrument :    Instrument #1
Sample Name: SS_56_Me
Misc Info  :
Vial Number: 10
```

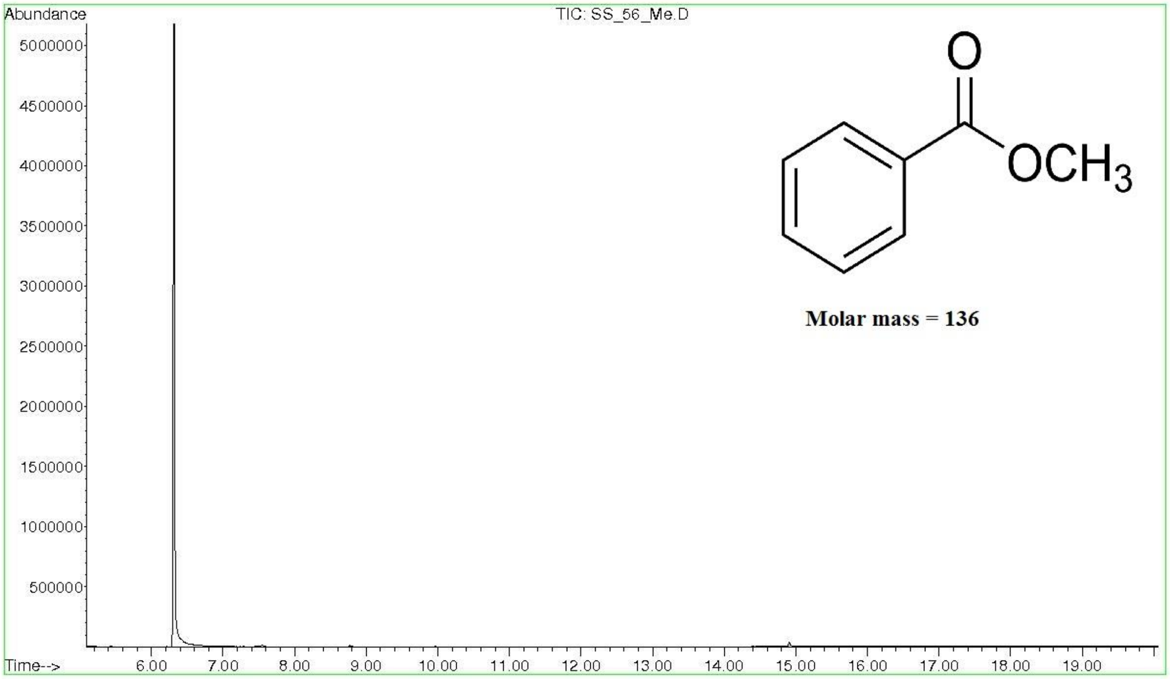

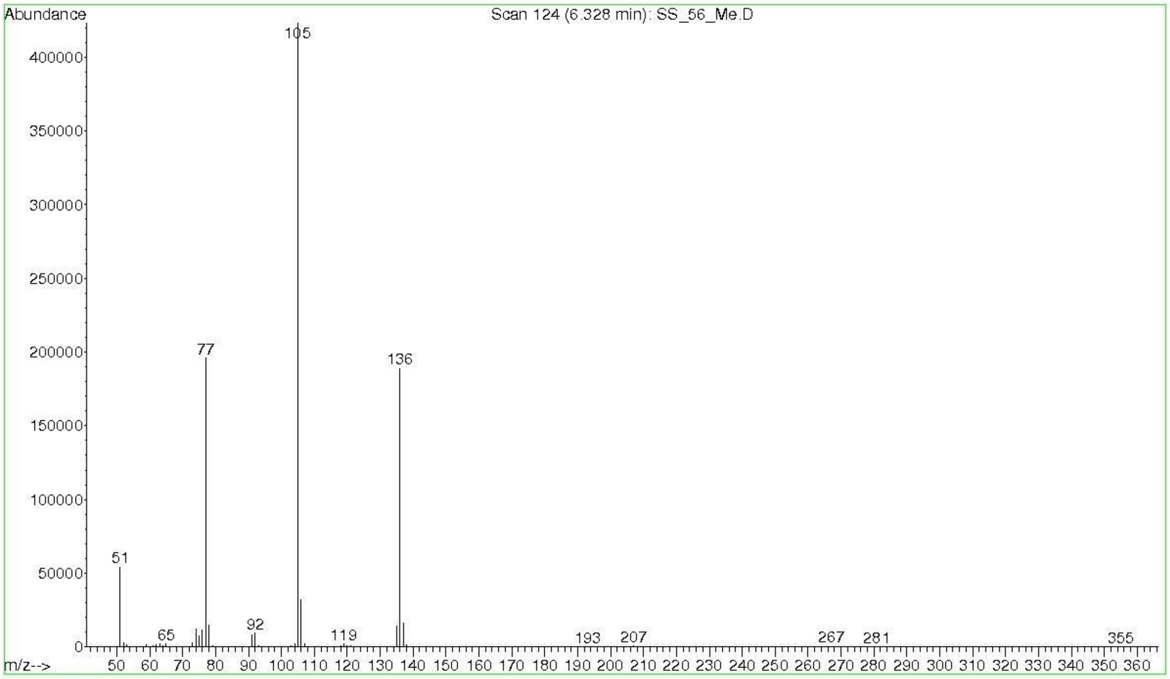

12. ^{1}H and ^{13}C NMR of the product

Methyl oleate

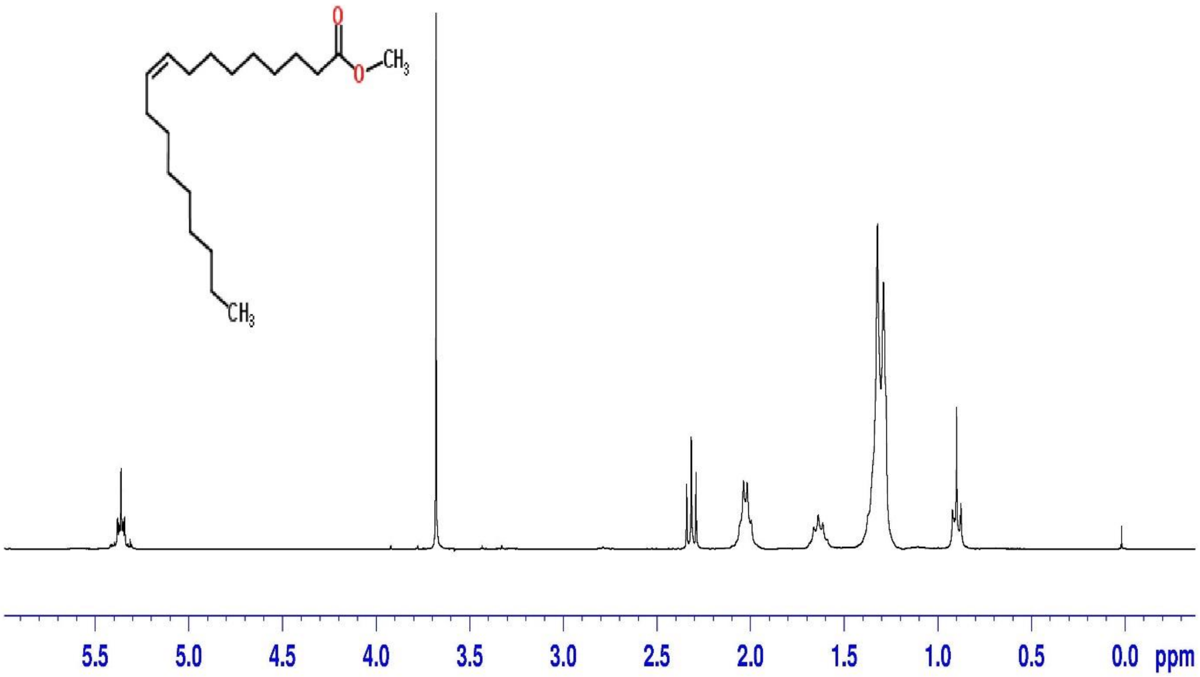

Oleic acid

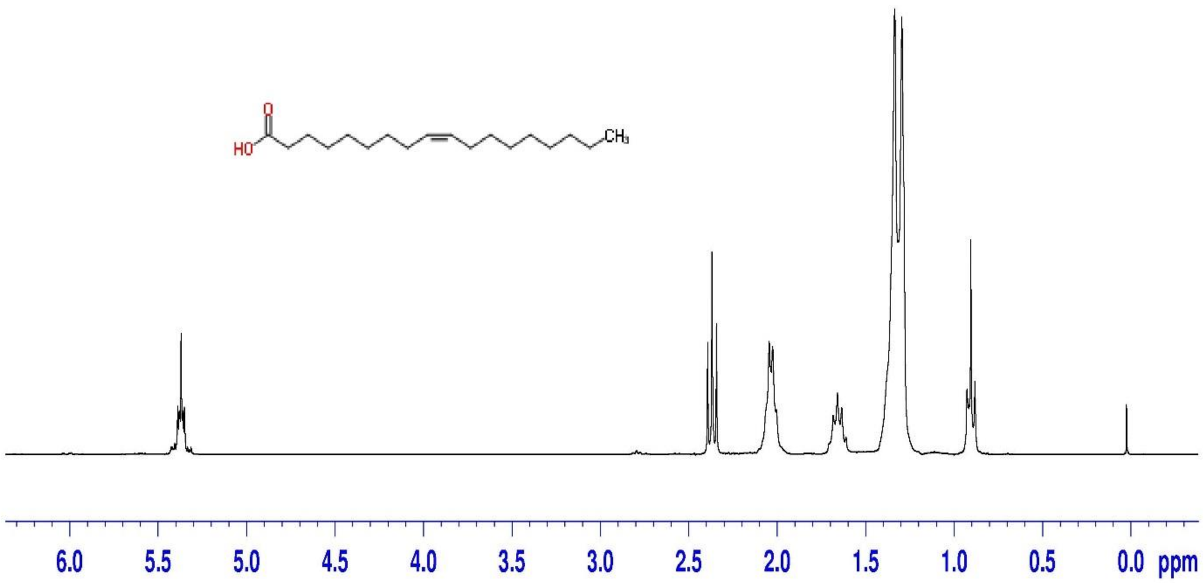

Methyl oleate

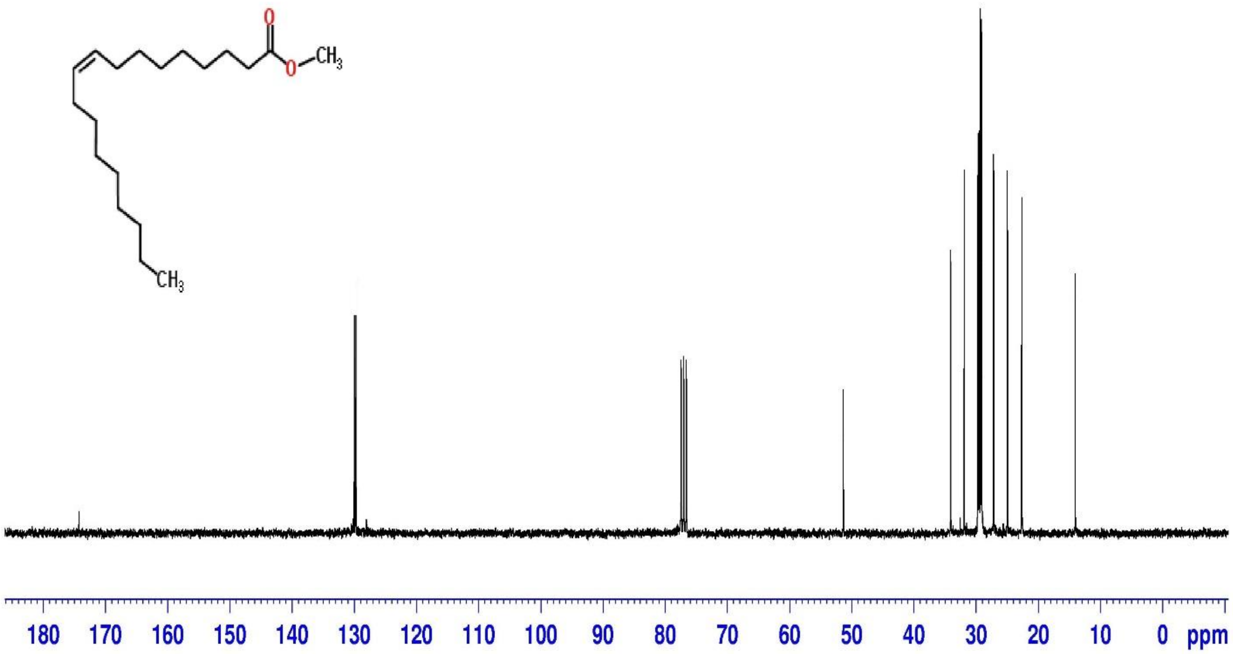

Oleic acid

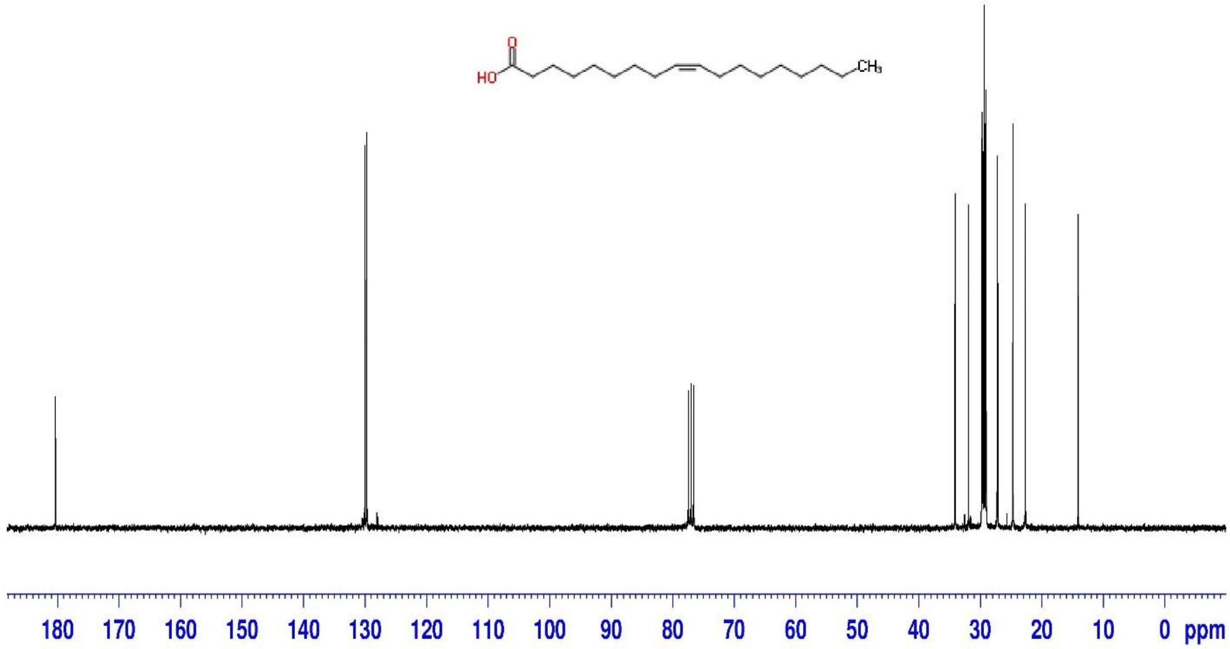

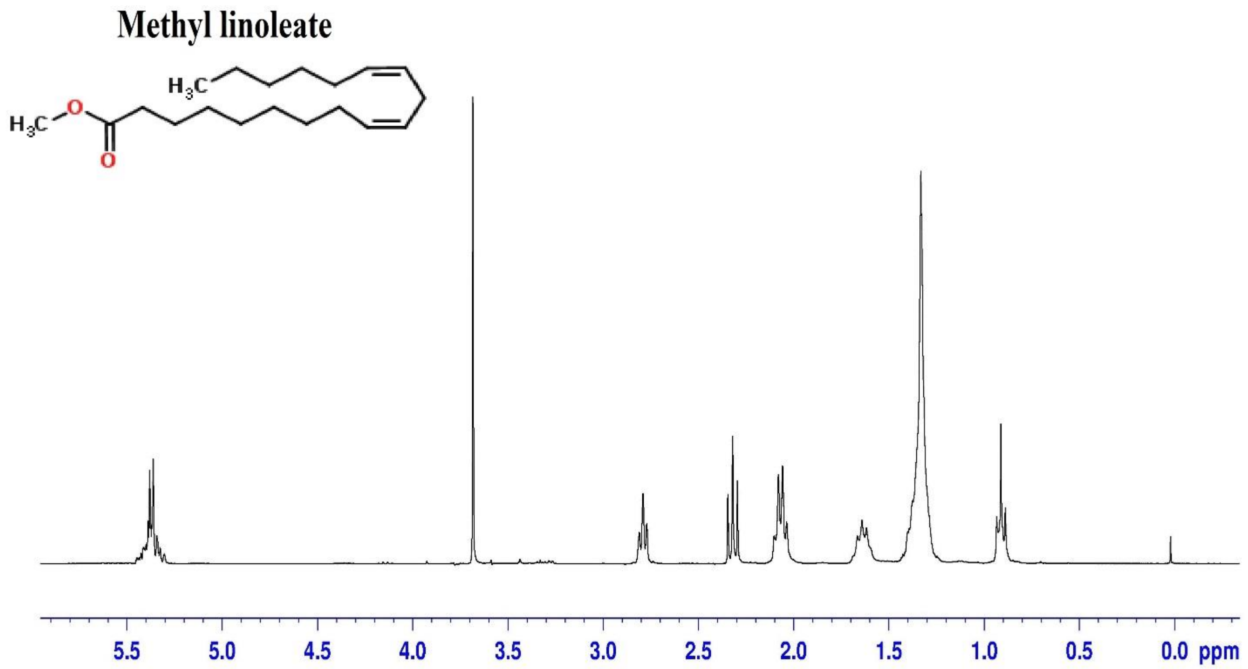
Methyl linoleate
H_3C
H_3C
O
O
5.5
5.0
4.5
4.0
3.5
3.0
2.5
2.0
1.5
1.0
0.5
0.0 ppm

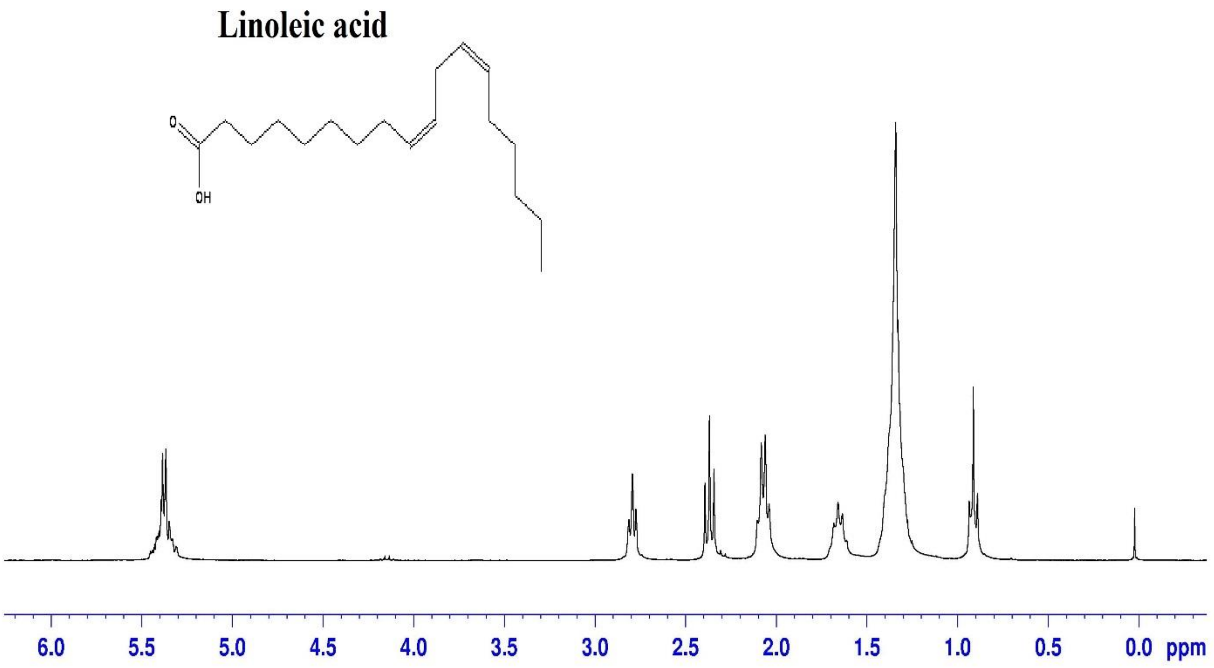
Linoleic acid
O
OH
6.0
5.5
5.0
4.5
4.0
3.5
3.0
2.5
2.0
1.5
1.0
0.5
0.0 ppm

Methyl linoleate

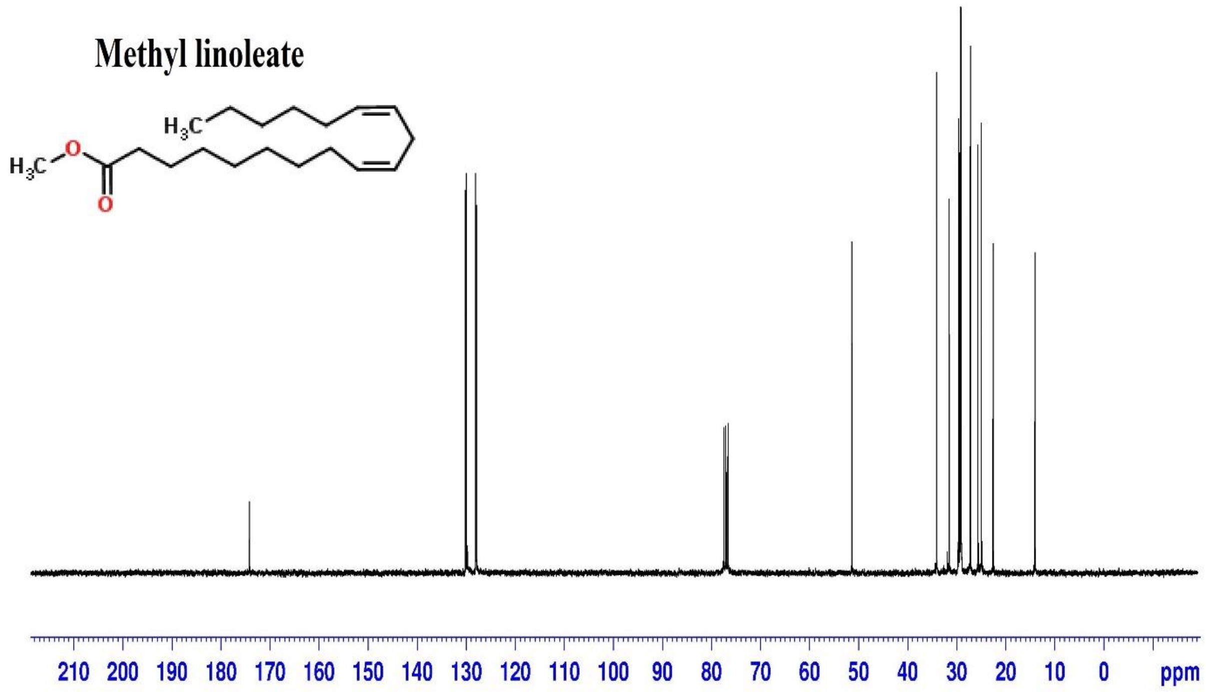

Linoleic acid

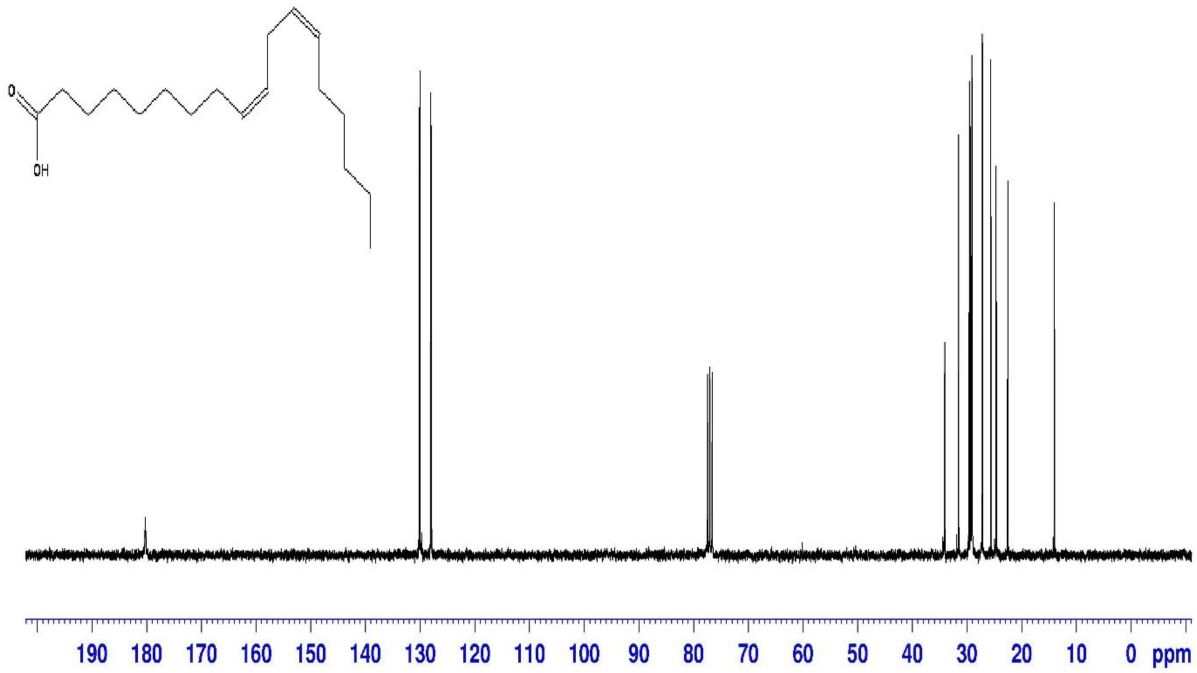

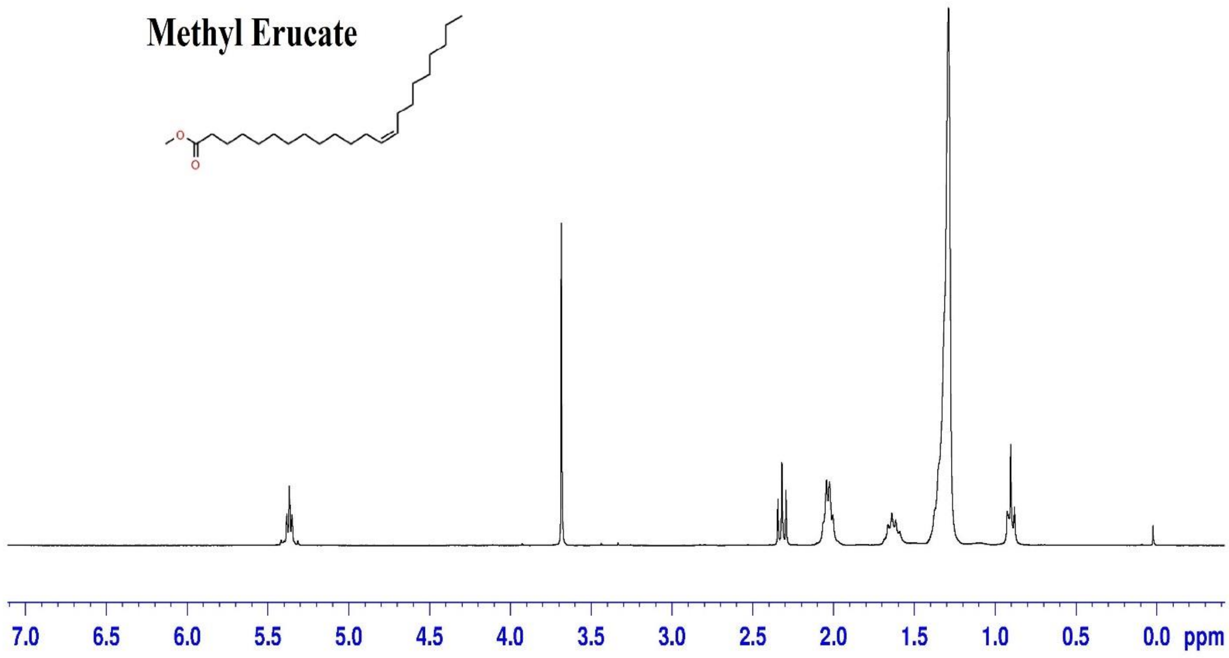
Methyl Erucate
O
O
7.0 6.5 6.0 5.5 5.0 4.5 4.0 3.5 3.0 2.5 2.0 1.5 1.0 0.5 0.0 ppm

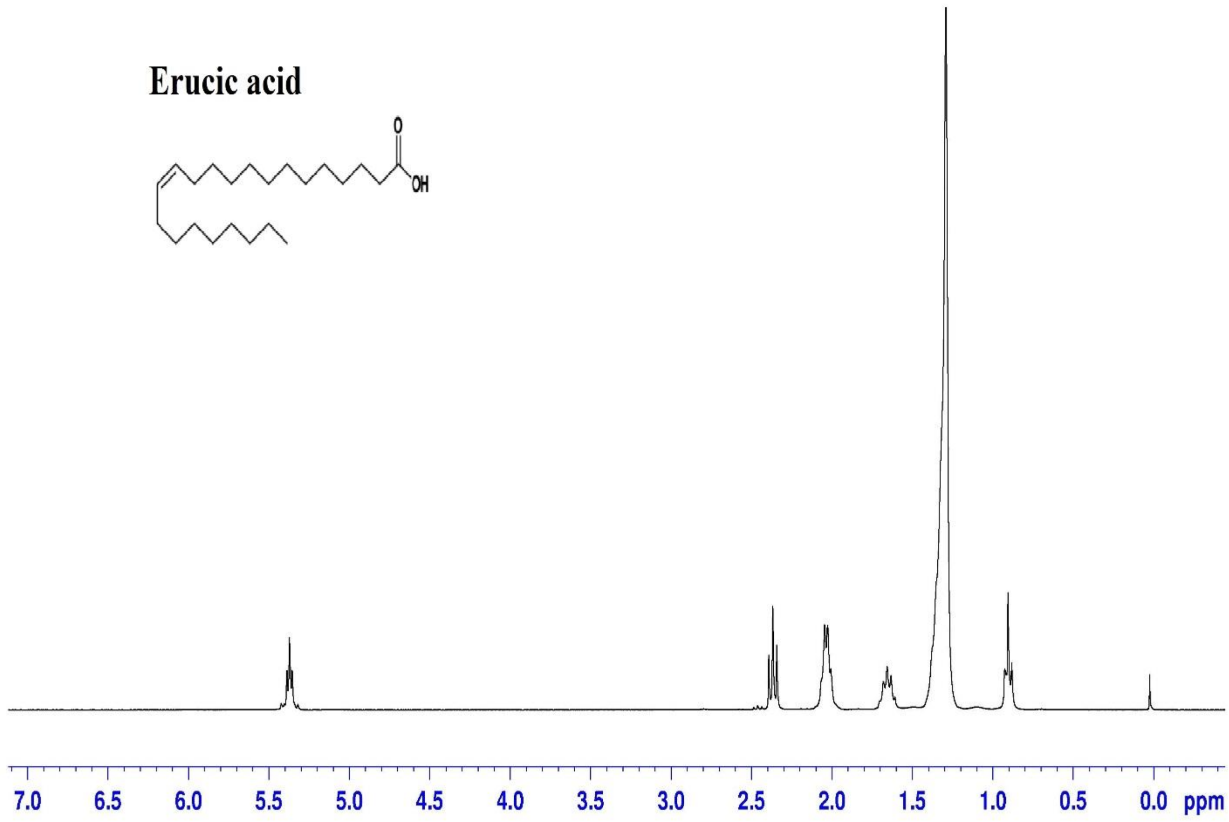
Erucic acid
O
OH
7.0 6.5 6.0 5.5 5.0 4.5 4.0 3.5 3.0 2.5 2.0 1.5 1.0 0.5 0.0 ppm

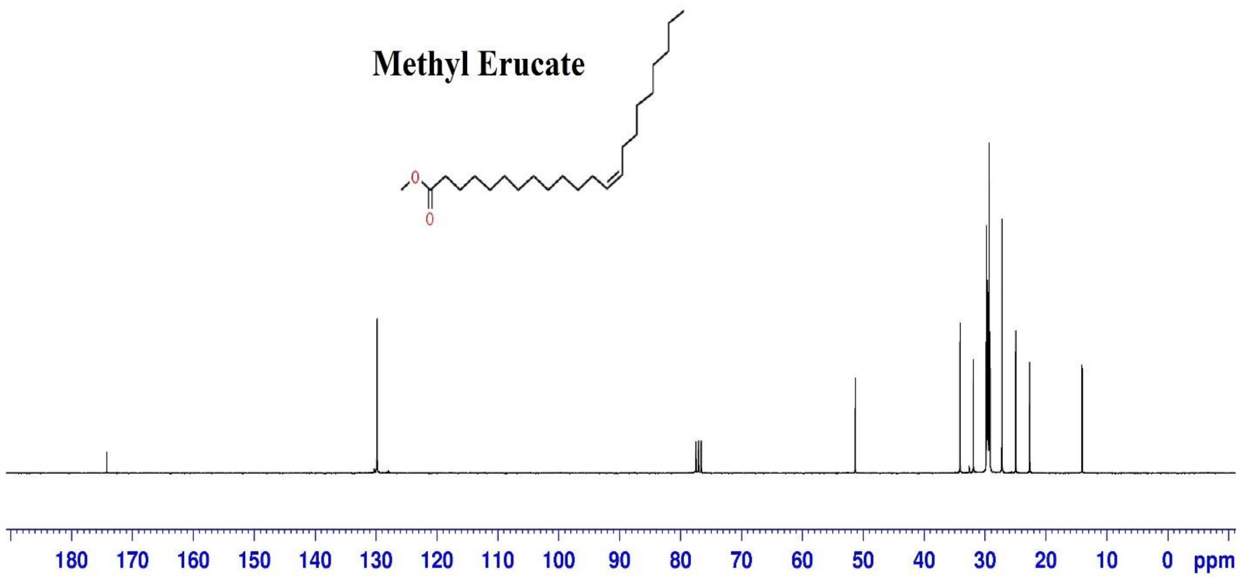
Methyl Erucate
O
O
180 170 160 150 140 130 120 110 100 90 80 70 60 50 40 30 20 10 0 ppm

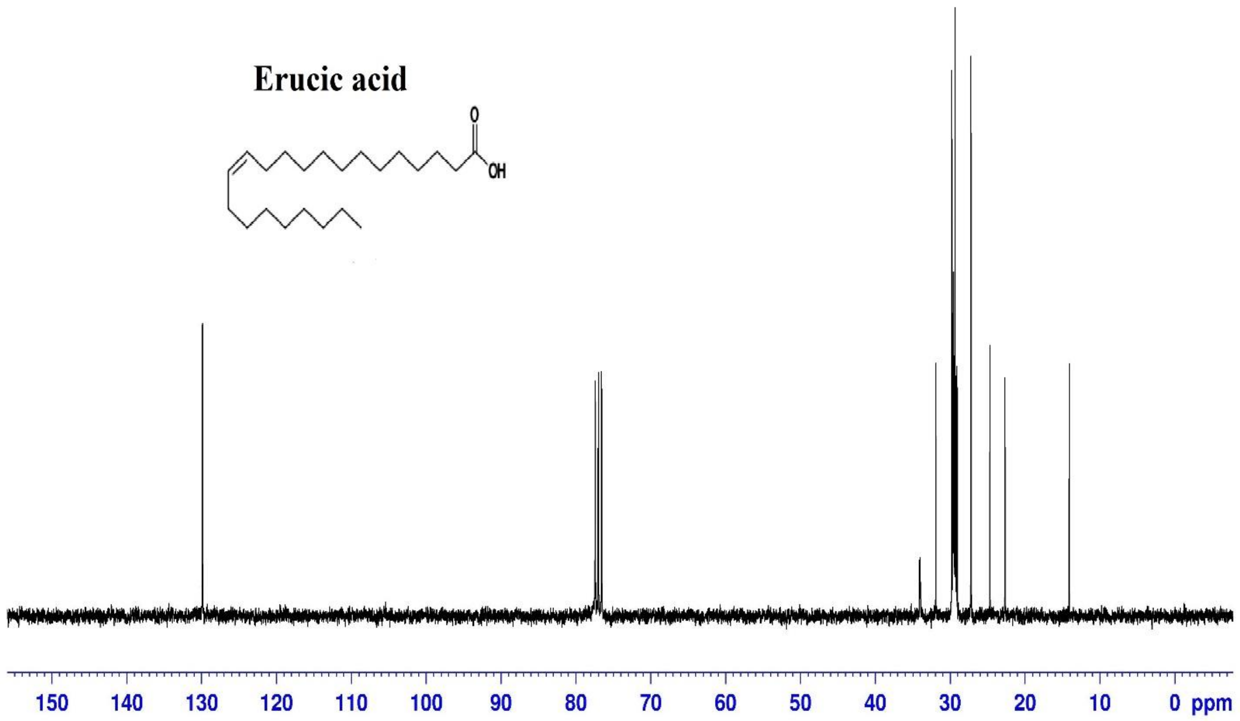
Erucic acid
O
OH
150 140 130 120 110 100 90 80 70 60 50 40 30 20 10 0 ppm

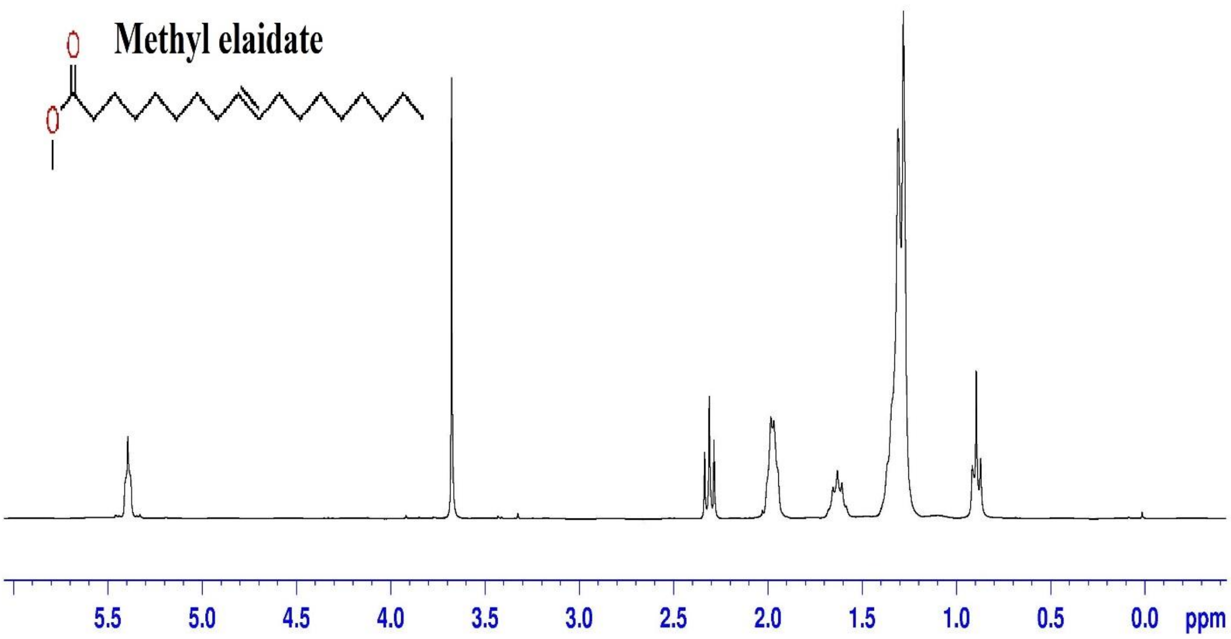
Methyl elaidate
O
O
5.5
5.0
4.5
4.0
3.5
3.0
2.5
2.0
1.5
1.0
0.5
0.0
ppm

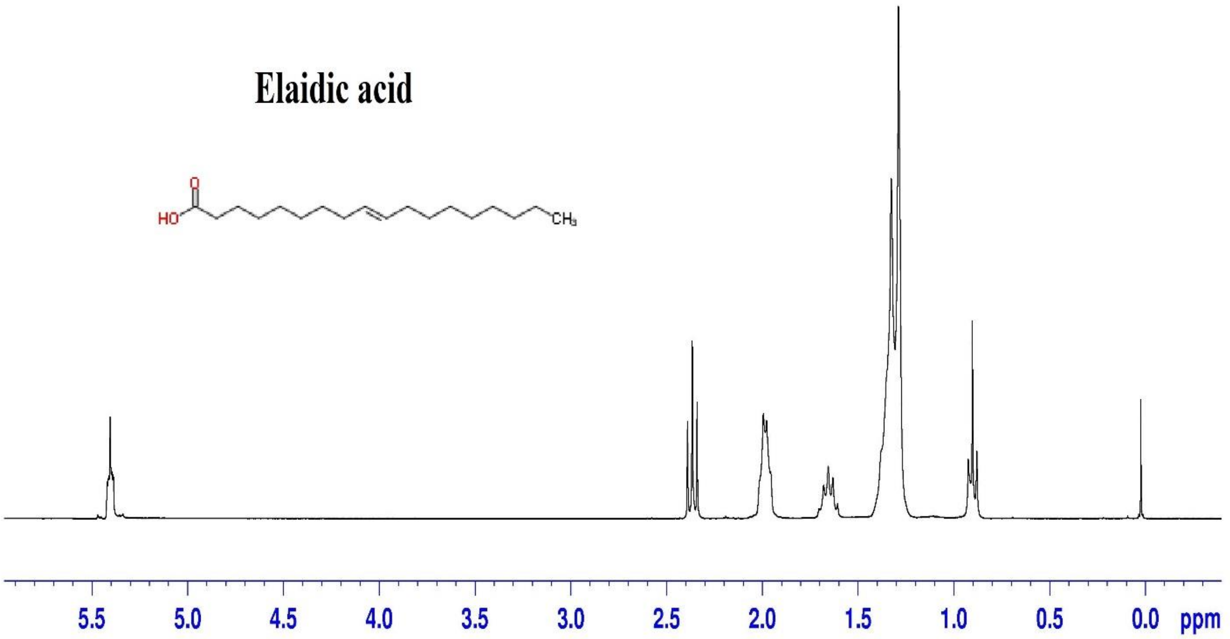
Elaidic acid
HO
O
CH3
5.5
5.0
4.5
4.0
3.5
3.0
2.5
2.0
1.5
1.0
0.5
0.0
ppm

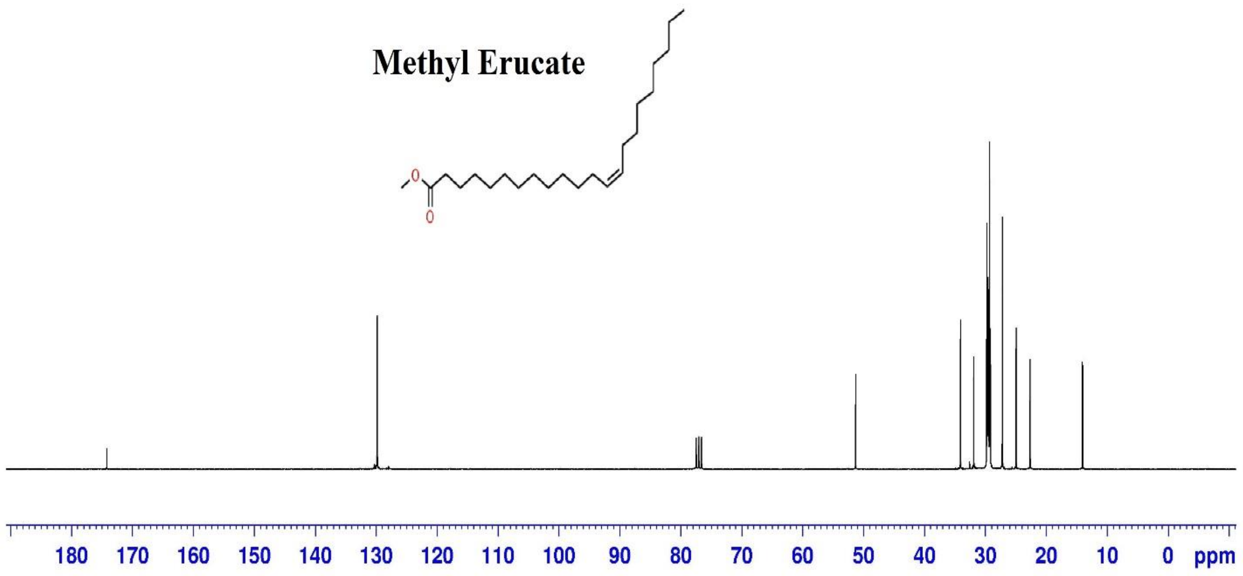
Methyl Erucate
O
O
180 170 160 150 140 130 120 110 100 90 80 70 60 50 40 30 20 10 0 ppm

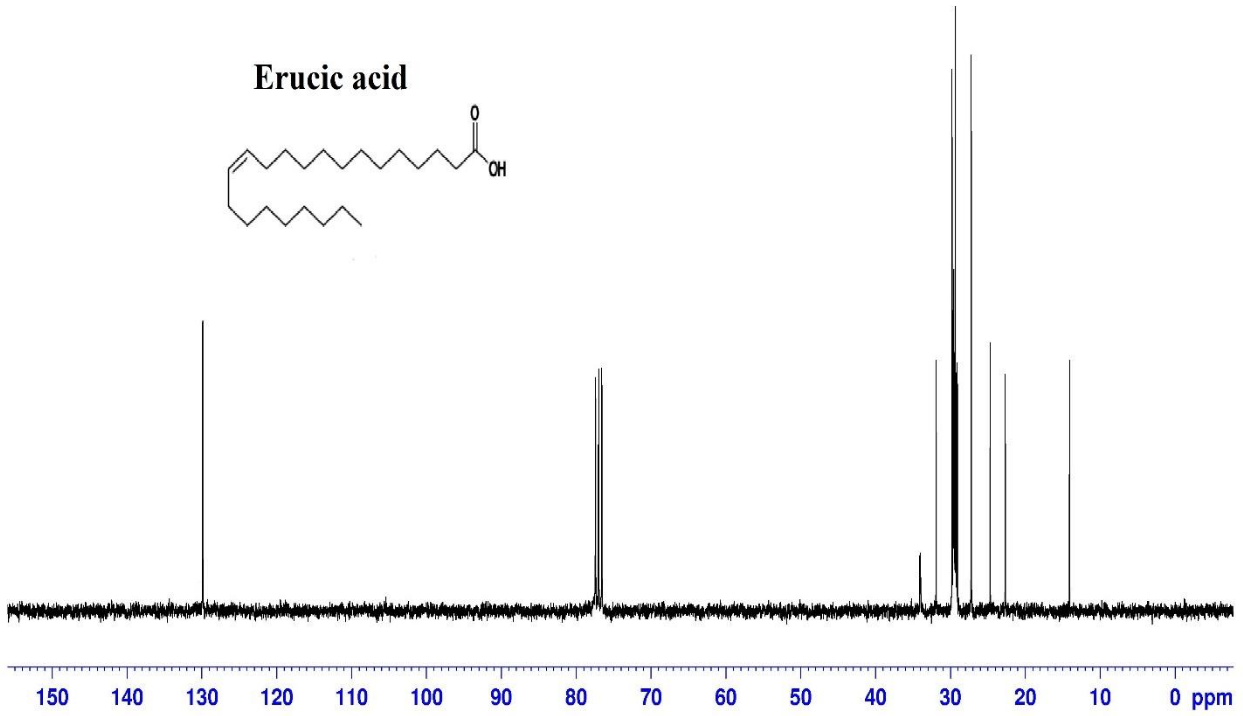
Erucic acid
O
OH
150 140 130 120 110 100 90 80 70 60 50 40 30 20 10 0 ppm
